# Recommendations for the prevention, screening, diagnosis, staging, and management of cervical cancer in areas with limited resources: Report from the International Gynecological Cancer Society consensus meeting

**DOI:** 10.3389/fonc.2022.928560

**Published:** 2022-08-18

**Authors:** Fernando Cotait Maluf, Graziela Zibetti Dal Molin, Andreia Cristina de Melo, Eduardo Paulino, Douglas Racy, Robson Ferrigno, Pedro Luiz Serrano Uson Junior, Reitan Ribeiro, Renato Moretti, Jose Carlos Sadalla, Angelica Nogueira-Rodrigues, Filomena Marino Carvalho, Glauco Baiocchi, Donato Callegaro-Filho, Nadeem R. Abu-Rustum

**Affiliations:** ^1^ Hospital Beneficiência Portuguesa (BP) Mirante, São Paulo, Brazil; ^2^ Hospital Israelita Albert Einstein, São Paulo, Brazil; ^3^ Instituto Nacional de Câncer-Inca, Rio de Janeiro, Brazil; ^4^ Oncomed, Rio de Janeiro, Brazil; ^5^ HCOR Oncologia, São Paulo, Brazil; ^6^ Instituto de Oncologia do Paraná, Paraná, Brazil; ^7^ Hospital Sírio Libanês, São Paulo, Brazil; ^8^ Universidade Federal de Minas Gerais, Minas Gerais, Brazil; ^9^ Faculdade de Medicina da USP, São Paulo, Brazil; ^10^ A.C. Camargo Cancer Center, São Paulo, Brazil; ^11^ Memorial Sloan Kettering Cancer Center, New York, NY, United States

**Keywords:** cervical cancer, radiotherapy, chemotherapeutics, limited resource area, limited resource countries

## Abstract

**Introduction:**

Nearly 85% of cervical cancer new cases are diagnosed in limited resources countries. Although several strategies have been proposed to reduce the disease burden, challenges remain to provide the best possible care. We report recommendations from an expert consensus meeting convened to address from prevention to management of cervical cancer in limited resources countries.

**Methods:**

The expert panel, composed by invited specialists from 38 developing countries in Africa, Asia, Eastern Europe, Latin America, and the Middle East, convened in Rio de Janeiro in September 2019, during the Global Meeting of the International Gynecological Cancer Society (IGCS). Panel members considered the published scientific evidence and their practical experience on the topics, as well as the perceived cost-effectiveness of, and access to, the available interventions. The focus of the recommendations was on geographic regions rather than entire countries because medical practice varies considerably in the countries represented. Resource limitation was qualified as limited access to qualified surgeons, contemporary imaging or radiation-oncology techniques, antineoplastic drugs, or overall funding for provision of state-of-the-art care. Consensus was defined as at least 75% of the voting members selecting a particular answer of the multiple-choice questionnaire, whereas the majority vote was considered as 50% to 74.9%.

**Results:**

Consensus was reached for 25 of the 121 (20.7%) questions, whereas for 54 (44.6%) questions there was one option garnering between 50% to 74.9% of votes (majority votes). For the remaining questions, considerable heterogeneity in responses was observed.

**Discussion:**

The implementation of international guidelines is challenging in countries with resource limitations or unique health-care landscapes. The development of guidelines by the health care providers in those regions is more reflective of the reality on the ground and may improve medical practice and patient care. However, challenges remain toward achieving that goal at political, economic, social, and medical levels.

## Introduction

Cervical cancer is the fourth most common malignancy among females, both for incidence and mortality. It is estimated that nearly 570.000 new cases and 310.000 deaths worldwide each year (1). The burden of cervical cancer is disproportionately distributed between low-/middle-income countries (LMICs) and high-income countries (HICs). Whereas the incidence of cervical cancer and its mortality have decreased by nearly 75% over the past 50 years in most HICs, around 85% of new cases of this disease are diagnosed in LMICs (2, 3). Improvements in HICs have been ascribed mostly to the use of pap test screening and the ability to diagnose and treat patients with pre-invasive lesions, whereas low population coverage, poor-quality cytology, incomplete follow-up of screen-positive women, and barriers to effective treatment are potentially responsible for the low success of cervical-cancer prevention programs in LMICs (2, 4).

More than 80% of women followed over time will be exposed to at least one high-risk variance of Human Papilloma Virus (HPV). The HPV vaccination as a preventive strategy should target young people before initiation of sexual activity, focusing on girls and boys aged 10–14 years. Moreover, the availability of HPV vaccination has led to an even brighter future for women in HICs to reduce the burden of cervical neoplasia (5). HPV vaccination offers at the same time the potential to decrease the incidence and mortality of cervical neoplasia, but at the same time highlights the disparities in cervical cancer prevention if vaccines are not available due to socioeconomic factors, especially in low-income countries, depending on the coverage of its implementation (6–8). Although considerable progress has been made in Latin America, Africa, Asia, Eastern Europe and the Middle East, specific obstacles to widespread adoption of HPV vaccination have been highlighted and include limited awareness of HPV disease, the vaccine, safety, costs, and cultural barriers (9).

Although several strategies have been proposed aiming to reduce the burden of cervical cancer in LMICs (5, 7, 10), challenges remain for the practicing physician to provide the best possible care in areas with limited resources and with varying national health-care policies. This is the first article reporting the recommendations from an expert consensus meeting convened to address the challenges on prevention, screening, diagnosis, staging, and management of cervical cancer in areas with limited resources. The meeting was convened under the auspices of the International Gynecological Cancer Society.

## Methods

### Panel organization, composition, and objectives

**Figure 1:**
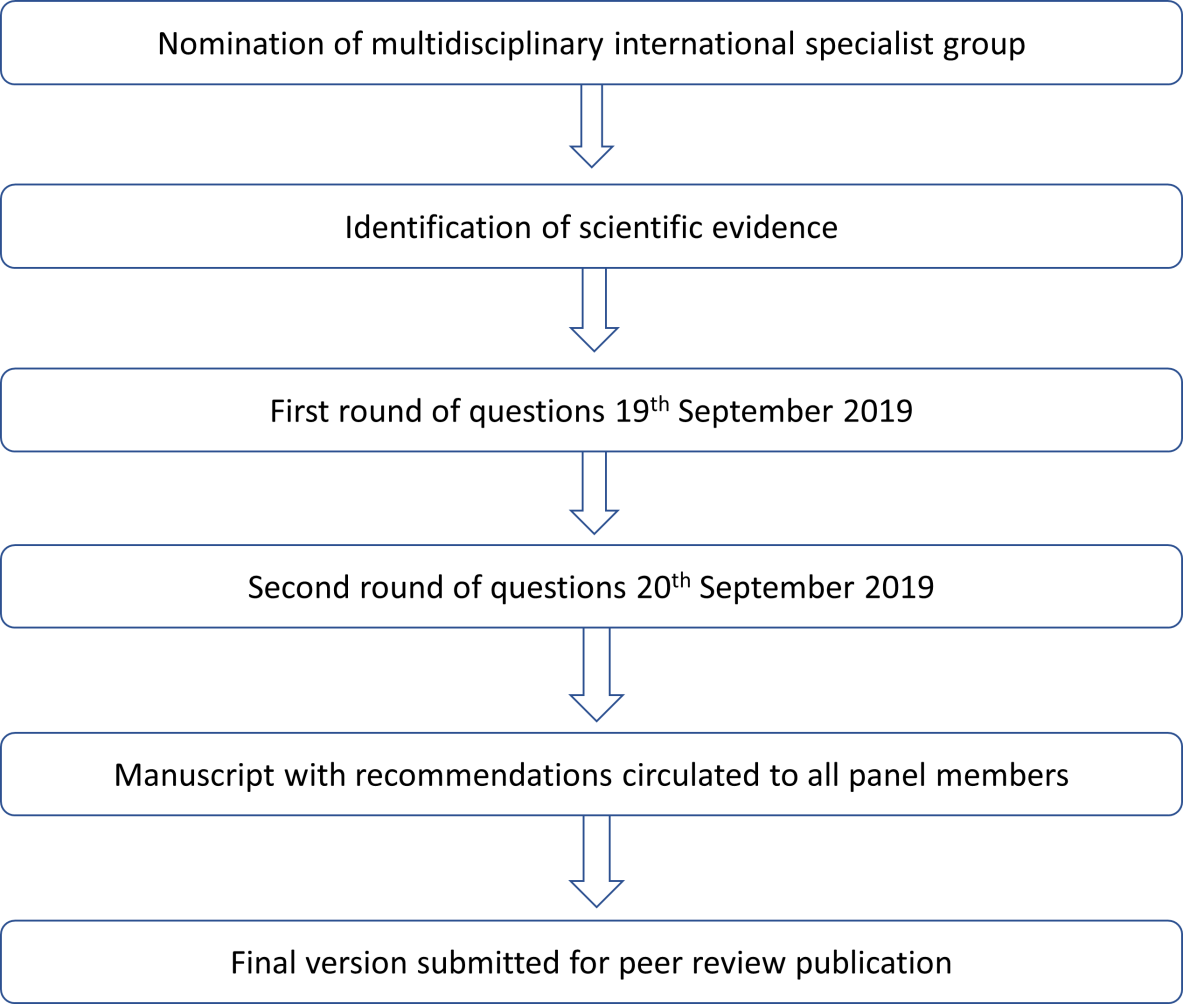
Development process.

The questions addressed by the panel were proposed by a 15-member committee as the most relevant for decision-making in areas facing resource limitations. The panel, composed of invited specialists in gynecological oncology from 38 developing countries in Africa, Asia, Eastern Europe, Latin America, and the Middle East, aimed to provide recommendations on salient issues that affect the management of cervical cancer in these areas (**Supplementary Table 1**). The panel was composed by physicians who are opinion leaders for the treatment of gynecological malignancies in gynecology, surgery, medical oncology, radiation oncology, radiology, and pathology in their respective countries. The panel provided the recommendations using an electronic voting system in sessions held on 19th and 20th September 2019, during the Global Meeting of the International Gynecological Cancer Society, convened in Rio de Janeiro, Brazil (**Figure 1**). To provide such recommendations, panel members considered the published scientific evidence and their practical experience on the topics, as well as the perceived cost-effectiveness of, and access to, the available interventions. One polling session with multiple-choice questions was scheduled for each of the main topics that constitute the subheadings described below. When answering each multiple-choice question, panel members were instructed to consider that their recommended intervention was approved and available, with no contraindications in the scenario described by the corresponding question. Moreover, recommendations were to be given for non-frail patients (defined as having an Eastern Cooperative Oncology Group [ECOG] performance status between 0 and 2) and for patients with squamous cell carcinoma or adenocarcinoma of the cervix. Finally, the staging classification used throughout was the latest version 2018 provided by the International Federation of Obstetrics and Gynecology (11).

### Definition of resource limitation

Despite the World Bank’s classification of economies into four income groups (high, upper-middle, lower-middle, and low (12)), and notwithstanding the fact that the panel includes members from countries that may belong to different income groups, the socioeconomic framework used during the discussions and reported herein relates to the availability of ideal resources. This is particularly relevant in some of the countries represented, which have heterogeneous health-care systems. In Brazil, for example, significant disparities exist in health care; although this remains the responsibility of the federal government, care is in fact provided in two major systems (public and private) which display very diverse characteristics in terms of access to state-of-the art care. This is particularly evident in oncology, given the high costs associated with providing health care in the public system, the sole provider for nearly 75% of the Brazilian population (13). The same situation affects other countries represented by the panel, whereas some of the countries have a more uniform, albeit constrained, health-care system. Regardless of the situation in individual countries, the focus of the current work and recommendations is on “area” rather than “country”, under the assumption that medical practice may not be necessarily constrained in a whole country and still be subject to resource limitation in given areas or settings within a country. Finally, resource limitation was qualified as the limited access to qualified surgeons, contemporary imaging or radiation-oncology techniques, antineoplastic drugs, or overall funding for provision of state-of-the-art care.

### Statistical analysis

Results are presented descriptively for each of the questions addressed by the panel. Consensus was reached if at least 75% of the voting members selected a particular answer, not considering in the denominator of this proportion the response option “unqualified to answer” (**Table 1**). On the other hand, the response option “abstains” (used when a member felt impeded to provide a qualified response for reasons other than lack of knowledge, including the presence of conflicts of interest or absence of a reasonable response option) was considered in the denominator. In accordance with the journal’s guidelines, we will provide our data for the reproducibility of this study in other centers if such is requested.

**Table 1:** Voting consensus.

Session	Majority voting	Consensus
Session 1 Prevention, screening, diagnosis, staging, and surveillance of cervical cancer	* Two vaccine doses, separated by 6 months, under the age of 15 for boys and girls. * Bethesda classification is the preferred classification for cervical cytology. * Yearly cervical cytology followed by testing every 3 years after two consecutive normal exams. * Stopping screening in women aged 65 years with evidence of two adequate negative prior screening results. * IHC is necessary for suspected adenocarcinoma, sarcoma, neuroendocrine or rare tumors.	* Colposcopy is only indicated in cases with HSIL (CIN2/3) or higher cytological findings. * Histopathological report for surgical specimens should include information on margins, tumor size and grade, depth of invasion, lymph vascular and perineural invasion, mitotic index, necrosis, parametrium involvement, and lymph-node metastasis. * Recommended staging method is abdominal and pelvic computed tomography (CT) plus chest X-ray for those with clinical stages FIGO IB2 to IVA. * Recommended follow-up is every 3 months in the first 2 years, and every 6 months thereafter until 5 years from treatment.
Session 2 Treatment of early-stage cervical cancer	* For stage IA2 cervical cancer is recommended radical hysterectomy when no fertility is desired, conization for similar diagnosis in women desiring to preserve fertility. * For women with stage IB1-IB2 cervical cancer, surgery alone is recommended for areas in which RDT is not available. In areas where surgeons do not have a full training in gynecology oncology, chemoradiation should be recommended. * For women with cancer confined to the cervix with a clinically visible tumor >4 cm (stage IB3) to IIA, chemoradiation alone is recommended. * Follow up is recommended after an incidental diagnosis of stage IA2 disease without lymph vascular invasion in a simple hysterectomy specimen in areas in which surgeons do not have full training in gynecology oncology. * Conventional external RDT is the recommended technique as the minimum required treatment for women with early-stage cervical cancer who need adjuvant RDT. * In institutions with only cobalt machines, patients with early-stage cervical cancer can be treated with external RDT. * Vaginal vault brachytherapy after external radiotherapy, as a boost, for patients with early-stage cervical cancer and at least two intermediate-risk features.	* For women with stage IA2 cervical cancer wishing to preserve fertility, trachelectomy is the treatment recommendation indicated by panel members. * Neoadjuvant chemotherapy followed by surgery is indicated for women with cancer confined to the cervix with a clinically visible tumor >4 cm (stage IB3) to IIA in areas in which RDT is not available. * Chemoradiation alone is recommended for women with cancer confined to the cervix with a clinically visible tumor >4 cm (stage IB3) to IIA in areas in which surgeons do not have full training in gynecology oncology. * Neoadjuvant chemotherapy followed by simple hysterectomy was recommended for women with cancer confined to the cervix with a clinically visible tumor >4 cm (stage IB3) to IIA in areas in which surgeons do not have full training in gynecology oncology and RDT is not available. * Open surgery was indicated as the recommended approach for patients with stage IB-IIA cervical cancer undergoing radical hysterectomy. * For women with early-stage cervical cancer undergoing surgery and having at least one high-risk feature (positive surgical margins, pathologically involved pelvic nodes, or positive involvement of the parametria), adjuvant RDT and chemotherapy should be indicated. * Both primary and adjuvant external RDT can be administered to women with early-stage cervical cancer in institutions where there are only conventional radiotherapy techniques.
Session 3 Locally advanced cervical cancer	* RDT alone can be indicated when chemotherapy is not available in a timely manner for patients with locally advanced disease. * In terms of external RDT technique for stages IB3 through IVA disease, the minimal recommended option is conventional radiation. Cobalt machines is appropriate if it is the only external technique available. * RDT with chemotherapy is appropriate if no brachytherapy is available for patients with stages IIB through IVA disease. * When radiotherapy is not available, neoadjuvant chemotherapy followed by surgery in locally advanced disease is an option. * For patients not eligible to cisplatin, the recommended radiosensitizing agent is carboplatin. * Hysterectomy should not be recommended after chemoradiation for patients with bulky (>4 cm) tumors and no residual tumor after treatment.	* Primary concomitant chemoradiation is recommended for stages IIB to IVA cervical cancer. * Chemoradiation alone is recommended for patients with locally advanced disease in areas where surgeons do not have full training in gynecologic oncology, and for patients with HIV/AIDS or other forms of immunosuppression. * A two-dimensional conventional brachytherapy technique is recommended for eligible patients with stages IB3 through IVA disease after external radiation. * For women with suspected or pathologically confirmed para-aortic node involvement, primary chemoradiation with extended-field radiotherapy is recommended. * Weekly cisplatin is the preferred radiosensitizing agent for the general patient population and for patients with HIV/AIDS or other forms of immunosuppression.
Session 4 Treatment and clinical complications of metastatic or recurrent cervical cancer	* The recommended first-line treatment for patients with platinum-naïve metastatic or recurrent cervical cancer not amenable to salvage loco-regional treatment when all resources are available is a regimen of cisplatin, paclitaxel, and bevacizumab. * When resources are limited, the recommended first-line treatment for such patients is cisplatin plus paclitaxel. * The recommended first-line treatment for AIDS and other immunosuppressed patients not amenable to salvage loco-regional treatment in areas with limited resources is full-dose platinum-based chemotherapy doublet. * When monotherapy is indicated as the first line with a non-platinum option, paclitaxel should be recommended. * The best intervention to control vaginal bleeding secondary to tumor progression in a patient previously treated with radiotherapy is vaginal packing with or without tranexamic acid. * Percutaneous nephrostomy is recommended as the best intervention to treat extrinsic ureteral compression secondary to tumor progression.	* For patients not amenable to salvage loco-regional treatment and not eligible to receive cisplatin, carboplatin plus paclitaxel should be the regimen of choice. * The best intervention to treat fecal incontinence due to rectovaginal fistula is surgical management by a diverting colostomy. * Sexual functioning appointments should be offered for cervical cancer survivors in the majority of patients. * Either paclitaxel or gemcitabine can be considered as appropriate treatment options for women with metastatic cervical cancer at any point according to its availability and lower price.

RDT, radiotherapy; IHC, Immunohistochemistry.

## Results

### Section 1 - Prevention, screening, diagnosis, staging, and surveillance of cervical cancer

There was no consensus for any of the questions related to HPV vaccination and screening in areas with limited resources (**Supplementary Table 2**, **3**). Regarding which HPV vaccine should be recommended, the quadrivalent vaccine was chosen by the largest percentage of panel members (42.1%), whereas 30.3% recommended any among the bivalent, quadrivalent and nonavalent vaccines. Despite the absence of consensus, 66.7% of panel members indicated a preference for two vaccine doses, separated by 6 months under the age of 15, with only 21.7% giving preference for three doses.

The Bethesda classification was the preferred classification for cervical cytology obtaining 72.2% of votes. In addition, at least 50% of voters recommended the following (1): an initial yearly cervical cytology followed by testing every 3 years after two consecutive normal exams (61.2% of voters) (2); stopping screening in women aged 65 years with evidence of two adequate negative prior screening results and no history of cervical intraepithelial neoplasia (CIN) of grade 2 or higher (59.3%); and (3) referral of patients with abnormal cytology to colposcopy followed by biopsy and treatment only if high grade squamous intraepithelial lesion (HSIL, i.e., CIN2/CIN3) or higher is confirmed on biopsy (52.6%). Nearly 40% of panel members believed HPV testing should be routinely available in areas with limited resources, whereas nearly a third recommended this practice only in selected cases, and 27.1% were against such testing. There was considerable variability in the opinion about the ideal age at which screening should begin for sexually active women.

Consensus was reached for two questions related to the diagnosis of cervical cancer in areas with limited resources (**Supplementary Table 4**): colposcopy is only indicated in cases with HSIL (CIN2/3) or higher cytological findings, and the histopathological report for surgical specimens should include information on margins, tumor size and grade, depth of invasion, lymph vascular and perineural invasion, mitotic index, necrosis, parametrium involvement, and lymph-node metastasis. Most of the votes was obtained for the need to have immunohistochemistry studies of suspected adenocarcinoma, neuroendocrine carcinoma, sarcoma, or rare tumors (72.0% of votes) and for considering that cervical cytology (positive for carcinoma) is insufficient for diagnosing clinically suspicious tumors (59.3%).

Regarding the diagnostic methods required for staging patients with cervical cancer, 80% of respondents recommended abdominal and pelvic computed tomography (CT) plus chest X-ray for those with early clinical stages (FIGO IB2 and 3). Likewise, 85.2% of panel members recommended these exams for patients with clinical stages II-IVA (**Supplementary Table 5**). For two questions, there was at least one recommendation made by at least 50% of voters (abdominal and pelvic CT plus chest X-ray for clinical stage IB1 or earlier cervical cancer, and abdominal and pelvic magnetic resonance imaging (MRI) plus chest X-ray for this same setting when trachelectomy is being considered.

Over 80% of panel members indicated their preference for follow-up every 3 months in the first 2 years, and every 6 months thereafter until 5 years from treatment. Slightly over 60% of voters were in favor of vaginal cytology in the follow-up of patients with early-stage disease undergoing radical hysterectomy, whereas 58.4% recommended follow-up every 3 months in the first 2 years, and every 6 months thereafter until 5 years from treatment in patients early-stage disease undergoing curative treatment. For the other questions, there was considerable heterogeneity in responses (**Supplementary Table 6**).

### Recommendations based on consensus:

* Colposcopy is only indicated in cases with HSIL (CIN2/3) or higher cytological findings.
* Histopathological report for surgical specimens should include information on margins, tumor size and grade, depth of invasion, lymph vascular and perineural invasion, mitotic index, necrosis, parametrium involvement, and lymph-node metastasis.
* Recommended staging method is abdominal and pelvic computed tomography (CT) plus chest X-ray for those with clinical stages FIGO IB2 to IVA.
* Recommended follow-up is every 3 months in the first 2 years, and every 6 months thereafter until 5 years from treatment.

### Section 2 - Treatment of early-stage cervical cancer

There was consensus for seven (24.1%) of the 29 questions related to the treatment of early-stage cervical cancer in areas with limited resources (**Supplementary Table 7**). For women with stage IA2 cervical cancer wishing to preserve fertility, trachelectomy was the treatment recommendation indicated by 77.6% of panel members. For women with cancer confined to the cervix with a clinically visible tumor >4 cm (stage IB3) to IIA in areas in which radiotherapy is not available, 75.6% of panelists indicated neoadjuvant chemotherapy followed by surgery. For a similar patient in areas in which surgeons do not have full training in gynecology oncology, chemoradiation alone was recommended by 81.4% of panelists. If, conversely, neither radiotherapy is available nor do surgeons have full training in gynecology oncology in a given area, neoadjuvant chemotherapy followed by simple hysterectomy was recommended by 75.8% of panelists; in this case, however, 19.2% of panelists abstained from voting. Open surgery was indicated as the recommended approach by 95.2% of panel members in cases of patients with stage IB-IIA cervical cancer undergoing radical hysterectomy. For women with early-stage cervical cancer undergoing surgery and having at least one high-risk feature (positive surgical margins, pathologically involved pelvic nodes, or positive involvement of the parametria), adjuvant radiotherapy and chemotherapy was recommended by 80.0% of panelists. Finally, 94.0% of panel members indicated that both primary and adjuvant external radiotherapy can be administered to women with early-stage cervical cancer in institutions where there are only conventional radiotherapy techniques.

There was a majority vote for 11 (37.9%) questions related to the treatment of early-stage cervical cancer in areas with limited resources (**Supplementary Table 7**). For women with stage IA2 cervical cancer, 65.7% of voters recommended radical hysterectomy when no fertility is desired, whereas 67.5% recommended conization for similar diagnosis in women desiring to preserve fertility. For women with stage IB1-IB2 cervical cancer, surgery alone was recommended by 63.4% of panelists for areas in which radiotherapy is not available. For similar patients in areas where surgeons do not have a full training in gynecology oncology, 61.2% of panelists recommended chemoradiation, whereas 14.9% recommended radiation alone. For women with cancer confined to the cervix with a clinically visible tumor >4 cm (stage IB3) to IIA, chemoradiation alone was recommended by 70.0% of panel members. After an incidental diagnosis of stage IA2 disease without lymph vascular invasion in a simple hysterectomy specimen, and absence of enlarged pelvic lymph nodes evaluated by computed tomography scan, the best course of action in an area without qualified surgeons in gynecologic oncology is strict follow-up in the opinion of 69.7% of voters. Conventional (2-dimension) external radiotherapy is the recommended technique as the minimum required treatment for women with early-stage cervical cancer who need adjuvant radiotherapy according to 64.0% of voters. In institutions with only cobalt machines, patients with early-stage cervical cancer can be treated with external radiotherapy in the opinion of 72.5% of panelists. On the other hand, 64.9% of panelists do not recommend adjuvant vaginal vault brachytherapy alone instead of external radiotherapy for patients with early-stage cervical cancer and at least two intermediate-risk features (lymph vascular invasion, cervical stromal invasion, or tumor size ≥4 cm). Conversely, 65.0% of panelists recommend vaginal vault brachytherapy after external radiotherapy, as a boost, for patients with early-stage cervical cancer and at least two intermediate-risk features. Finally, 59.0% of panelists always recommend vaginal vault brachytherapy after external radiotherapy, as boost, for patients with early-stage cervical cancer and at least one high-risk feature (positive surgical margins, pathologically involved pelvic nodes, or positive involvement of the parametria); it should be noted, however, that an additional 34.4% of voters indicated they restrict this recommendation to patients with positive vaginal margins.

For the other 11 (37.9%) questions, there was considerable heterogeneity in responses from panel members. For some questions, there were two or more options sharing the vote in a relatively balanced manner. That was the case for questions related to the treatment of stage IB1-IB2 cervical cancer in general (with 40.8% of votes for surgery alone and 30.4% for surgery followed by radiation with or without chemotherapy); stage IB1-IIA disease in frail patients (41.5% of votes for chemoradiation and 32.1% for radiotherapy alone); early stages of cervical cancer after surgery with at least two intermediate-risk features (48.5% of votes for adjuvant radiotherapy alone and 43.3% for adjuvant radiotherapy and chemotherapy); for patients with cervical cancer scheduled for radical hysterectomy and pelvic lymphadenectomy in whom a suspicious lymph node is found at the beginning of the surgery (44.1% of votes for proceeding with surgery as planned and 42.3% for resecting the suspicious lymph node and performing lymphadenectomy and radical hysterectomy if it is confirmed positive by frozen section); and whether radical trachelectomy should be proposed in stage IB1 cervical cancer if trained surgeons are not available (49.6% of votes in favor, but with the patient referred to another service, and 41.2% of votes against radical trachelectomy). For some questions, there was a predominant answer achieving less than 50% of votes, with the remainder of votes distributed evenly among other options. Such was the case, for example, for neoadjuvant chemotherapy followed by surgery for stage IB1-IIA disease when radiotherapy is not available, and surgeons do not have adequate training in gynecological oncology.

### Recommendations based on consensus:

* For women with stage IA2 cervical cancer wishing to preserve fertility, trachelectomy is the treatment recommendation indicated by panel members.
* Neoadjuvant chemotherapy followed by surgery is indicated for women with cancer confined to the cervix with a clinically visible tumor >4 cm (stage IB3) to IIA in areas in which radiotherapy is not available.
* Chemoradiation alone is recommended for women with cancer confined to the cervix with a clinically visible tumor >4 cm (stage IB3) to IIA in areas in which surgeons do not have full training in gynecology oncology.
* Neoadjuvant chemotherapy followed by simple hysterectomy was recommended for women with cancer confined to the cervix with a clinically visible tumor >4 cm (stage IB3) to IIA in areas in which surgeons do not have full training in gynecology oncology and radiotherapy is not available.
* Open surgery was indicated as the recommended approach for patients with stage IB-IIA cervical cancer undergoing radical hysterectomy.
* For women with early-stage cervical cancer undergoing surgery and having at least one high-risk feature (positive surgical margins, pathologically involved pelvic nodes, or positive involvement of the parametria), adjuvant radiotherapy and chemotherapy should be indicated.
* Both primary and adjuvant external radiotherapy can be administered to women with early-stage cervical cancer in institutions where there are only conventional radiotherapy techniques.

### Section 3 - Locally advanced cervical cancer

There was consensus for eight (33.3%) of the 24 questions related to the treatment of locally advanced cervical cancer in areas with limited resources (**Supplementary Table 8**). Primary concomitant chemoradiation was recommended for stages IIB through IIIA (86.1% of votes), and IIIB, IIIC and IVA cervical cancer (90.4%), by most panel members. Chemoradiation alone was recommended by 86.5% of voters in patients with locally advanced disease in areas where surgeons do not have full training in gynecologic oncology, and by 79.6% of voters in the case of patients with HIV/AIDS or other forms of immunosuppression. In terms of the brachytherapy technique recommended for eligible patients with stages IB3 through IVA disease after external radiation, a two-dimensional conventional technique was indicated by 80.5% of voters. For women with suspected or pathologically confirmed para-aortic node involvement, primary chemoradiation with extended-field radiotherapy was recommended by 86.1% of voters. Finally, weekly cisplatin is the preferred radiosensitizing agent both in general (78.7%) and in patients with HIV/AIDS or other forms of immunosuppression (94.3%).

There was a majority vote for 11 (45.6%) questions posed to the panel (**Supplementary Table 8**). Radiotherapy alone was chosen by 72.1% of voters when chemotherapy is not available in a timely manner for patients with locally advanced disease. In terms of external radiotherapy technique for stages IB3 through IVA disease, the minimal recommended option was conventional radiation (64.1% of votes) compared with only 35.9% for conformal radiation. In institutions with only conventional radiotherapy, this was considered appropriate for all stages by 69.3% of panelists. Likewise, if cobalt machines were the only external technique available, it was considered appropriate for all stages by 70.7% of voters. If no brachytherapy is available, external-beam radiotherapy with chemotherapy was considered appropriate by 67.0% of voters for patients with stages IIB through IVA disease. For women with stages IB3 through IVA disease treated with primary chemoradiation or radiotherapy alone, the maximal accepTable duration of radiotherapy (whole-pelvic irradiation plus brachytherapy or external-beam boost) was 7 weeks for 50.7% of voters. When radiotherapy is not available, 71.4% of voters recommended neoadjuvant chemotherapy followed by surgery in locally advanced disease. For patients not eligible to cisplatin, the recommended radiosensitizing agent was carboplatin for 73.8% of panelists. Hysterectomy should not be recommended after chemoradiation for patients with bulky (>4 cm) tumors and no residual tumor after treatment, according to 66.3% of voters. In patients with locally advanced disease and poor geriatric score and/or poor performance status, radiation alone was recommended by 50.9% of voters. If radiotherapy is not available for such patients, best-supportive care was recommended by 59.3% of panelists.

For the other five (20.8%) questions, there was more heterogeneity in responses from panel members. In two cases, however, there was a predominant response approaching 50% of votes. In areas where no brachytherapy is available, external-beam radiotherapy with chemotherapy was recommended by 49.5% of panelists in the case of stages IB3 through IIA, whereas carboplatin plus paclitaxel was the choice of 49.4% of voters when neoadjuvant chemotherapy is indicated. For the other three questions, there was more heterogeneity in responses and a clear lack of a dominant option.

### Recommendations based on consensus:

* Primary concomitant chemoradiation is recommended for stages IIB to IVA cervical cancer.
* Chemoradiation alone is recommended for patients with locally advanced disease in areas where surgeons do not have full training in gynecologic oncology, and for patients with HIV/AIDS or other forms of immunosuppression.
* A two-dimensional conventional brachytherapy technique is recommended for eligible patients with stages IB3 through IVA disease after external radiation.
* For women with suspected or pathologically confirmed para-aortic node involvement, primary chemoradiation with extended-field radiotherapy is recommended.
* Weekly cisplatin is the preferred radiosensitizing agent for the general patient population and for patients with HIV/AIDS or other forms of immunosuppression.

### Section 4 – Treatment and clinical complications of metastatic or recurrent cervical cancer

There was consensus for only one (4.2%) of the 24 questions related to the treatment of metastatic or recurrent cervical cancer (**Supplementary Table 9**). This question related to the first-line treatment of patients not amenable to salvage loco-regional treatment and not eligible to receive cisplatin, for which 76.1% of voters recommended carboplatin plus paclitaxel. On the other hand, a majority vote was present for 13 (54.2%) questions posed to the panel. The recommended first-line treatment for patients with platinum-naïve metastatic or recurrent cervical cancer not amenable to salvage loco-regional treatment when all resources are available is a regimen of cisplatin, paclitaxel, and bevacizumab in the opinion of 69.2% of panelists. When resources are limited, the recommended first-line treatment for such patients is cisplatin plus paclitaxel according to 60.7% of voters. For patients with prior platinum (>6 months earlier) therapy, 57.4% indicated cisplatin plus paclitaxel when resources are limited. For patients with platinum therapy within the previous 6 months, 51.9% indicated carboplatin plus paclitaxel for areas with limited resources. The recommended first-line treatment for AIDS and other immunosuppressed patients not amenable to salvage loco-regional treatment in areas with limited resources is full-dose platinum-based chemotherapy doublet in the opinion of 66.7% of panelists. When monotherapy is indicated as the first line with a non-platinum option, paclitaxel was recommended by 71.6% of panelists. If there is no access to taxane or cost-limited resources for this drug, cisplatin plus fluorouracil was recommended by 61.8% of panelists. Salvage surgery alone was the recommended treatment option for a resecTable loco-regional recurrence without suspicion of lymph-node involvement in patients with comorbidities and/or contra-indication to cisplatin who were previously treated with radiation therapy in 54.7% of cases. If cisplatin is contra-indicated as a radiosensitizing agent, carboplatin is the recommended option for a resecTable loco-regional lymph-node recurrence in the opinion of 70.7% of panelists. For similar patients without comorbidities but treated initially only with surgery, chemoradiation with cisplatin was indicated by 59.1% of voters. Finally, the indication of best supportive care is the presence of an Eastern Cooperative Oncology Group performance status >2 in the opinion of 51.9% of voters, considering women with previously treated metastatic cervical cancer and with no access to a clinical trial. For the remaining 10 (41.7%) questions shown in **Supplementary Table 9**, there was more heterogeneity in responses, even if for some of these questions there was one predominant option garnering more votes (in some cases, close to half of the votes).

Eight of the questions presented to the panel were related to the management of clinical complications often seen in metastatic or recurrent cervical cancer. Consensus answers were given for two (25.0%) of those questions. Surgical management by diverting colostomy and colostomy bags was considered by 92.6% of panelists as the best intervention to treat fecal incontinence due to rectovaginal fistula. Likewise, 78.8% of panelists indicated they would recommend sexual functioning appointments for cervical cancer survivors in the majority of patients. In addition, there were three (37.5%) questions with a majority vote. The best intervention to control vaginal bleeding secondary to tumor progression in a patient previously treated with radiotherapy was vaginal packing with or without tranexamic acid in the opinion of 74.7% of panelists. Best supportive care was the best intervention to control pelvic pain secondary to tumor progression in a patient previously treated with radiotherapy according to 59.7% of voters. Percutaneous nephrostomy was recommended by 74.3% of voters as the best intervention to treat extrinsic ureteral compression secondary to tumor progression. For the other three questions, there was more substantial heterogeneity in responses.

The third group of questions related to drugs used in cervical cancer included in the World Health Organization (WHO) essential medicines list that can be purchased at an affordable price from generic manufacturers. Among those seven drugs, only paclitaxel and gemcitabine were considered as appropriate treatment options for women with metastatic cervical cancer by at least 75% of panelists. Moreover, there was a majority vote that ifosfamide, methotrexate and vinorelbine are not appropriate in this setting, whereas fluorouracil and topotecan are appropriate.

### Recommendations based on consensus:

* For patients not amenable to salvage loco-regional treatment and not eligible to receive cisplatin, carboplatin plus paclitaxel should be the regimen of choice.
* The best intervention to treat fecal incontinence due to rectovaginal fistula is surgical management by a diverting colostomy.
* Sexual functioning appointments should be offered for cervical cancer survivors in the majority of patients.
* Either paclitaxel or gemcitabine can be considered as appropriate treatment options for women with metastatic cervical cancer at any point according to its availability and lower price.

## Discussion

To our knowledge, this is the first consensus meeting, and the first attempt to provide wide-ranging recommendations for cervical cancer, involving specialists from a large number of countries that face resource limitations to screen for and treat cervical neoplasia. Although the major goal of the current initiative was not to obtain consensus for each question addressed by the panel, consensus is a desirable feature that was defined a priori as at least 75% of valid responses. However, consensus was reached for only 25 (20.7%) of the 121 questions presented to the panel described here, whereas for 54 questions there was one option garnering between 50% and 74.9% of votes. Therefore, for nearly 45% of all questions presented to the panel, there was considerable heterogeneity in responses, and no consensus could be reached. On the other hand, the very low percentages of abstentions and of voters who considered themselves as “unqualified to answer” suggests that the topics chosen are relevant in current practice and that panel members indeed have specific and variable preferences for many of the clinical issues discussed. The extent to which lack of consensus for some of the questions was due to characteristics at the country level, such as specificities of the health-care system, has not been ascertained in the current work. Likewise, we cannot determine if some of the heterogeneity in responses reflects the varied professional background of the voting members.

It is well known that the implementation of international guidelines is challenging in countries with resource limitations or unique health-care landscapes, given that most of those guidelines come from North America and Western Europe (14, 15). One alternative for those countries is to follow guidelines adapted or stratified by resource availability from organizations, such as the National Comprehensive Cancer Network (NCCN) or the European Society of Gynecological Oncology/European Society for Radiotherapy and Oncology/European Society of Pathology (16, 17). For example, NCCN guidelines have been adapted to specific world regions, such as the Middle East and North Africa (18) or Asia (19), and usually within defined disease settings (18). Another option for individual countries facing resource limitation is to develop their own guidelines and consensus panels, a strategy that has been adopted, for example, in India (15, 20). In this current work, we have taken advantage of a large number of specialists from several countries gathering for an international meeting, in order to organize a panel that could provide consensus recommendations for topics previously identified as relevant in cervical and also in vulvar cancer (data not shown here). The topics addressed in this article pertain to prevention, screening, diagnosis, staging, surveillance and management of cervical cancer. Regardless of the preferred process to develop and implement disease-specific recommendations, both LMICs and HICs can benefit from recommendations by the World Health Organization, which often apply to specific issues in selected disease settings (21–23).

The results of this consensus have several limitations that are important to note. First, the definition of developing countries was determined by the real needs and restrictions of each country included in the consensus and faced by the experts in the field. Limitation of access to surgically trained gynecological professional, high quality radiotherapy machines and systemic regimens. In this consensus the World Bank`s economies classification was not used solely to identify the countries included. Second, some questions related to concepts somewhat validated in the literature did not reached consensus, demonstrating that the difficulties faced by this countries included not only access but other adverse issues including socio-economic and cultural barriers. However, despite of these limitations, a fairly number of questions reached majority voting (65-70%) or consensus (>75%), making this consensus a valuable tool for countries with limitations of resources presented in this report. Finally, prospective intervention strategies will be necessary to eradicate cervical cancer in low- and middle-low-income countries and regular consensus including those countries can serve as a first step for this process.

## Author contributions

FM, GM, AM, EP, DR, RF, PU, RR, RM, JS, AN-R, FC, GB, DC-F, and NA-R have done substantial contributions to the conception of the work. All participated in the acquisition, analysis and interpretation of data for the work. All authors participated in the drafting of the work and revising it critically for important intellectual content All the authors approve the publication of the content and agree to be accounTable for all aspects of the work in ensuring that questions related to the accuracy or integrity of any part of the work are appropriately investigated and resolved.

## Conflict of interest

The authors declare that the research was conducted in the absence of any commercial or financial relationships that could be construed as a potential conflict of interest.

## Publisher’s note

All claims expressed in this article are solely those of the authors and do not necessarily represent those of their affiliated organizations, or those of the publisher, the editors and the reviewers. Any product that may be evaluated in this article, or claim that may be made by its manufacturer, is not guaranteed or endorsed by the publisher.

## Abbreviations

HSIL, high grade squamous intra-epithelial lesion; CIN, cervical intraepithelial neoplasia; LEEP, loop electrosurgical excision procedure; LLETZ, large loop excision of the transformation zone.
