# Supplementary material for: Recommendations for the prevention, screening, diagnosis, staging, and management of cervical cancer in areas with limited resources: Report from the International Gynecological Cancer Society consensus meeting
Source: Front Oncol. 2022 Aug 18;12:928560. doi: 10.3389/fonc.2022.928560 (PMC9434552; doi:10.3389/fonc.2022.928560)
Supplement: Supplementary file 1 [file Table_1.docx]

**Supplementary Materials for “”Recommendations for the prevention, screening, diagnosis, staging, and management of cervical cancer in areas with limited resources: report from the International Gynecological Cancer Society Consensus Meeting**

**List of colleagues that voted on the cervical cancer consensus:**

| *Abraham Peedicayil, MD* |
| --- |
| *Adriana Bermudez, MD* |
| *Adriana Castelo C de Moura, MD* |
| *Ailma larre, MD* |
| *Aisha Mustapha, MBBS, MDRM, FMCOG^1^* |
| *Aknar Calabrich, MD^2^* |
| *Alessandro Buda, MD^3^* |
| *Alexandre da Costa, MD* |
| *Alexandre Pupo Nogueira, MD^4^* |
| *Amanda Karani, MD* |
| *Amilcar Barreta, MD^5^* |
| *Amr El-Shalakany, MSc, MD, FRCOG^6^* |
| *Ana Carolina de Rezende, MD^7^* |
| *Ana Carolina Falcão, MD^7^* |
| *Ana Carolina Matos de Queiroz, MD^8^* |
| *Ana Cecilia, MD* |
| *Analia Miele, MD* |
| *Andre Campana, MD* |
| *Andrea Massad Ribeiro, MD* |
| *Andrea Paiva Gadelha Guimaraes, MD* |
| *Andres Estremadoiro Vargas, MD* |
| *Angeles Nico, MD^9^* |
| *Areta Agostinho Rodrigues de Souza, MD ^10^* |
| *Asima Mukhopadhyay, MD^11^* |
| *Caetano da Silva Cardial, MD^12^* |
| *Candice Santos, MD* |
| *Celia Maria Pais Viegas, MD^13^* |
| *Cezar Angelo Alfredo Filho, MD* |
| *Cinthia Barbisan, MD* |
| *Cláudia Bessa Pereira Chaves, MD^14^* |
| *Clement Khoury, MD^15^* |
| *Cristiano de Pádua Souza, MD^16^* |
| *Dalva Guedes Arnaud, MD* |
| *David Atallah, MD, MSc^17^* |
| *Delzio Bicalho, MD* |
| *Dércia Idite Changule,MD^18^* |
| *Diama Vale, MD* |
| *Diocésio Alves Pinto de Andrade, MD^19^* |
| *Eloise Allen Marques de Oliveira, MD^20^* |
| *Erlon Gil , MD* |
| *Fábio Francisco Oliveira Rodrigues, MD^21^* |
| *Fernanda Damian , MD* |
| *Fernando Cordero, MD* |
| *Fernando de Souza Nobrega, MD^7^* |
| *Fuat Demirkiran, MD^22^* |
| *Gabriel Alfredo Crimi, MD^23^* |
| *Gabriela Norese, MD^24^* |
| *Geovanne Pedro Mauro, MD^25^* |
| *Gilmara Anne da Silva Resende, MD^26^* |
| *Gonzalo Medina Navía, MD ^27^* |
| *Guilherme Bicudo Barbosa, MD* |
| *Guilherme S. Accorsi, MD ^28^* |
| *Gustavo Focchi, MD* |
| *Gustavo Guitmann, MD* |
| *Henrique Helber, MD* |
| *Herbert Ives Barretto Almeida, MD^29^* |
| *Ian G Bambury MB.BS, DM^30^* |
| *Igor Austin , MD* |
| *Isabel Cristina Chulvis do Val, MD^32^* |
| *Jeancarllo, MD* |
| *João Siufi Neto, MD* |
| *Jorge Lyra, MD^33^* |
| *Joseph Bernard, Jr, MD^34^* |
| *Juliana Karassawa Helito , MD* |
| *Juliana Martins Pimenta, MD^35^* |
| *Juliano Rodrigues da Cunha, MD^36^* |
| *Julio Lau, MD^37^* |
| *Julio Teixeira , MD* |
| *Junfen Xu, MD^38^* |
| *Jurema Sales , MD* |
| *Ketheryn Almeida, MD* |
| *Larissa Müller Gomes, MD^39^* |
| *Larissa Sandon, MD* |
| *Leandro Santos A. Resende, MD* |
| *Lillian Morgado Leitão, MD* |
| *Lina Maria Caicedo, MD ^40^* |
| *Linda J Rogers, MD^41, 42^* |
| *Lucas Albuquerque, MD* |
| *Lucas Rios Torres , MD* |
| *Luis E. Medina Fernández, MD^43^* |
| *Luiza Maciel, MD* |
| *Lyliana Coutinho Resende Barbosa, MD^44^* |
| *Marcel Davi Loureiro de Melo , MD* |
| *Marcelo Simonsen, MD* |
| *Maria Carolina Szymanski de Toledo, MD* |
| *Maria Del Pilar Estevez Diz, MD^45^* |
| *Maria Eduarda Bittencourt Damasceno, MD* |
| *Maria Jimena Lange, MD^46^* |
| *Mariana Camargo G. Forghieri, MD ^47^* |
| *Marina Vasco , MD* |
| *Mauricio S. Abrao , MD* |
| *Michelle Almeida, MD* |
| *Mila Oliveira , MD* |
| *Monia Hechiche, MD^48^* |
| *Montassar Ghalleb, MD^49^* |
| *Muhieddine Seoud, MD, FACOG, FACS^50^* |
| *Natacha Phoolcharoen, MD* |
| *Ons Kaabia, MD^51^* |
| *Patricia Izetti , MD* |
| *Poliana Albuquerque Signorini, MD ^52^* |
| *Rachele Grazziotin Reisner, MD^53^* |
| *Raquel C.M. Fernandes , MD* |
| *Reena George, MD^54^* |
| *Renato José Affonso Junior, MD^55^* |
| *Ricardo dos Reis, MD^56^* |
| *Ricardo Pedrini Cruz, MD* |
| *Ricardo Rodrigues de Souza , MD* |
| *Rodrigo Alves Pinto, MD^57^* |
| *Rodríguez Yanina Anahi, MD^58^* |
| *Ronaldo Lúcio Rangel Costa , MD* |
| *Rose Anorlu, MD* |
| *Rossana Catão Zampronha, MD* |
| *Samantha Cabral, MD^45^* |
| *Selma Gadria, MD^59^* |
| *Selva Soledad Cuellar Murillo, MD^60^* |
| *Sergio M Lucchini, MD^61^* |
| *Shahana Pervin, MD^62^* |
| *Shalini Rajaram, MD^63^* |
| *Shylasree TS, MD* |
| *Simona Stolnicu, MD^64^* |
| *Suzana Arenhart Pessini, MD^65^* |
| *Tariane Foiato, MD* |
| *Thais de Almeida, MD* |
| *Thaís Xavier Nogueira de Souza^7^* |
| *Thiago Lins Almeida, MD^66^* |
| *Thomas Konney, MD* |
| *Timothy A.O. Oluwasola, MBBS, MSc, MSCI (Northwestern), FWACS, FMCOG, FACS ^67^* |
| *Tomas Pichlik, MD^61^* |
| *Tracey Adams, MD^62^* |
| *Ts Shylasree, MD* |
| *Vandré Cabral Gomes Carneiro, MD^68^* |
| *Vanessa Alvarenga-Bezerra, MD* |
| *Vinicius Canezin Galletto, MD^7^* |
| *Vinotha Thomas, MD* |
| *Volodymyr Artyomenko MD, PhD, DSc(Med)^69^* |
| *Yue Shi, MD* |

Affiliations:

1: Department of Obstetrics and Gynaecology, Ahmadu Bello University/Teaching Hospital, Zaria, Kaduna State, Nigeria

2: Clinica AMO, Brazil

3: Ospedale Michele e Pietro Ferrero, Gynecologic Oncology Division, Verduno, Cuneo, Italy

4: Hospital Sírio Libanês - São Paulo/SP, Brazil

5: Oncologia 22 de Outubro de Mogi-Mirim-SP, Brazil

6: Gynecological oncology, Department of obstetrics and gynecology, Ain Shams University, Cairo

7: Hospital Israelita Albert Einstein, SP, Brazil

8: Oncocentro Ceará/Fortaleza-CE , Brazil

9: Instituto de Oncología A. Roffo (Universidad de Buenos Aires), Argentina

10: Kora Saúde- Palmas/TO, Brazil

11: Kolkata Gynecology Oncology Trials and Translational Research Group, Chittaranjan National Cancer Institute, Kolkata, India

12: Departamento de ginecologia, FMABC, Brazil

13: Instituto Nacional de Câncer -Inca, RJ, Brazil

14: Division of Clinical Research and Technological Development, Brazilian National Cancer Institute, Rio de Janeiro, Brazil

15: Department of Radiation Oncology, Clemenceau Medical Center, Beirut, Lebanon

16: Hospital de Câncer de Barretos, Brazil

17: Saint Joseph University, Hôtel Dieu de France university Hospital, Beirut, Lebanon

18: Central Hospital of Maputo, Mozambique

19: InORP – Instituto Oncológico de Ribeirão Preto, Brazil

20: Serviço de Ginecologia e Mama - HOSPITAL ARAÚJO JORGE DA ACCG GOIÁS, Brazil

21: Serviço de Oncoginecolgia e Mastologia do Instituto de Câncer Dr Arnaldo Vieira de Carvalho , SP, Brazil

22: Istanbul university-cerrahpasa gynecologic oncology department Turkey

23: Gynecology and Obstetrics Department of CEMIC, a University Hospital  in Buenos Aires Argentina

24: Buenos Aires University Hospital, Argentina.

25: Instituto de Radioterapia do ABC, SP, Brazil

26: Fundação centro de controle de oncologia do Amazonas – FCECON, Brazil

27: Hospital San Juan De Dios Tarija-Bolivia

28: Faculdade de Medicina de Catanduva , Brazil

29: Universidade Federal da Bahia, Brazil

30: University Hospital of the West Indies, Jamaica

32: *Universidade Federal Fluminense, RJ, Brazil*

33: Surgical Oncology. Federal University of Maranhão – UFMA. Brazil

34: Medical Oncology, Innovating Health International Cancer Program, Port-au-Prince, Haiti

35: Hospital BP Mirante, R Martiniano de Carvalho 965, SP, Brazil

36: Universidade Federal de Uberlândia, MG, Brazil

37: Hospital General San Juan de Dios, Guatemala

38: Department of Gynecologic Oncology, Women’s Hospital, Zhejiang University School of Medicine, Hangzhou 310006, China

39: Centro Paulista de Oncologia - Oncoclínicas/SP, Brazil

40: Gynecologic Oncology , Bogota, Colombia

41: Department of Obstetrics and Gynaecology, Groote Schuur Hospital and the University of Cape Town, Cape Town, South Africa

42: SA MRC/UCT Gynaecological cancer Research Centre

43: Oncology center CEON, Arequipa PERÚ

44: Hospital das Clínicas Samuel Libânio, MG, Brazil

45: Instituto do Cancer do Estado de SP, ICESP, SP, Brazil

46: University Hospital, Buenos Aires, Argentina

47: Dep. Cirurgia de Alta Complexidade do H. M.Maternidade Escola Vila Nova Cachoeirinha, SP, Brazil

*48: Medicine school of Tunis,Tunis Manar University, Tunis Tunisia.*

49: Institute of oncology Tunis, Tunisia

50: Department of Obstetrics and Gynecology, American University Medical Centre, Beirut, Lebanon

51: Université de Sousse, Faculté de Médecine de Sousse, Hôpital Farhat Hached, Service de Gynécologie Obstétrique, 4000, Sousse, Tunisie

52: Fundação Centro de Controle de Oncologia do Amazonas – FCECON, Brazil

53: Instituto Nacional de Câncer -Inca, RJ, Brazil

54: Christian Medical College Vellore

55: Departamento de radioterapia, Hospital de Base Rio Preto/ Funfarme, Brazil

56: Hospital de Câncer de Barretos, Brazil

57: Oncologia DOR Pernambuco, Brazil

58: Hospital Marie Curie, Hospital Evita Pueblo , Argentina

59: Hannibal Medical Center, Tunisia

60: Instituto Oncologico A. Roffo, Argentina

61: Gynecology oncology section, Sanatorio Allende de Cordoba, Argentina

62: National Institute of Cancer Research & Hospital , Dhaka, Bangladesh

63: AIIMS Rishikesh, India

64: Department of Pathology, University of Medicine, Pharmacy, Sciences and Technology of Targu Mures, Romania

65: Universidade Federal do Rio Grande do Sul, Brazil

66: Oncoclinicas CPO/PB, Brazil

67: Gynaecologic Oncology Unit, Department of Obstetrics & Gynaecology, College of Medicine, University of Ibadan, and University College Hospital, Ibadan, Nigeria.

68: Hospital de Cancer de Pernambuco, SP, Brazil

69: Honoured Physician of the Ukraine, Ministry of Health of the Ukraine Expert, Department Obstetrics and Gynecology, Odessa National Medical University, Odessa, UKRAINE

**Supplementary Table 1: List of 38 countries represented by the panel**

Argentina, Bahamas, Bangladesh, Bolivia, Brazil, China, Colombia, Egypt, Ethiopia, Georgia, Ghana, Guatemala, Haiti, India, Indonesia, Italy, Jamaica, Jordan, Lebanon, Mexico, Mozambique, Nepal, Nigeria, Pakistan, Peru, Philippines, Qatar, Romania, Saudi Arabia, South Africa, Syria, Thailand, Tunisia, Turkey, Uganda, Ukraine, Venezuela, and Zambia

**Supplementary Table 2. Questions related to prevention of cervical cancer.**

| **Question** | **Answers and frequency of responses** | | | | | | | |
| --- | --- | --- | --- | --- | --- | --- | --- | --- |
| Which HPV vaccine should be recommended for cervix cancer prevention in an area with limited resources? | Bivalent | Quadrivalent | Nonavalent | Any of the above | Abstain | – | – | – |
|  | 16.5% | 42.1% | 11.2% | 30.3% | 0% | – | – | – |
| Which prophylactic HPV vaccination scheme (frequency and doses) for general population do you recommend in an area with limited resources? | 3 doses: 0, 2 and 6 months | 3 doses: 0, 6 and 60 months | 2 doses: 0 and 6 months for children under the age of 15 | 1 dose for children under the age of 15 | None | Abstain | – | – |
|  | 21.7% | 0% | 66.7% | 11.7% | 0% | 0% | – | – |
| Which is the recommended target population for HPV vaccination in an area with limited resources? | All men from 9 to 26 years, all women from 9 to 45 years and all HIV positive | All women from 9 to 45 years and all HIV positive | All men and women from 9 to 14 years and all HIV positive | All women from 9 to 14 years and all HIV positive | All men and women from 9 to 11 years and all HIV positive | All women from 9 to 11 years and all HIV positive | None | Abstain |
|  | 20.3% | 5.7% | 29.3% | 18.7% | 16.3% | 9.8% | 0% | 0% |

Answers to not all questions may total 100% due to rounding.

**Supplementary Table 3.** Questions related to screening of cervical cancer.

| **Question** | **Answers and frequency of responses** | | | | | | | |
| --- | --- | --- | --- | --- | --- | --- | --- | --- |
| Should HPV test be routinely available in areas with limited resources? | Yes | No | Just in special cases (cytologic-histologic discordance) | Abstain | – | – | – | – |
|  | 39.9% | 27.1% | 31.6% | 1.5% | – | – | – | – |
| Which is the recommended Pap smear classification in areas with limited resources? | Bethesda | Papanicolaou | Normal vs abnormal | None | Abstain | – | – | – |
|  | 72.2% | 17.3% | 9.0% | 0% | 1.5% | – | – | – |
| Who should collect the samples for cervical cancer screening by cytology (Pap smear) in an area with limited resources? | Gynecologist | Any physician | Nurse | Any qualified healthcare professional or the patient (self-collect) | Any of the above | None | Abstain | – |
|  | 7.5% | 13.4% | 6.0% | 44.8% | 27.6% | 0% | 0.8% | – |
| At which age should cervical cancer screening with cytology begin in sexually-active, HIV-negative women, in areas with limited resources? | 1 year after first sexual intercourse, regardless of age | Less than 3 years after first sexual intercourse, regardless of age | First sexual intercourse and/or age 18 years | First sexual intercourse and/or age 21 years | 25 years | 30 years | None | Abstain |
|  | 13.3% | 23.3% | 10.0% | 13.3% | 29.2% | 9.2% | 0.8% | 0.8% |
| Which technique is the most adequate for cervical cancer screening in areas with limited resources? | Pap smear | Co-testing (combined Pap and HPV testing) | Visual inspection with acetic acid or Lugol’s iodine | Visual inspection with acetic acid and HPV test | None/  Abstain | - | – | – |
|  | 46.7% | 18.5% | 10.4% | 19.3% | 5.1% |  |  |  |
| What is the best Pap smear periodicity in a non-immunocompromised population in areas with limited resources? | Every year | Every 3 years | Every 5 years | Every year; but after 2 normal exams, should be done every 3 years | Every year; after 2 normal exams, every 5 years | Once every 10 years from age 21-60 years | None | Abstain |
|  | 11.5% | 15.8% | 5.8% | 61.2% | 4.3% | 0.7% | 0.7% | 0% |
| When should cervical cancer screening be stopped in a non-immunocompromised population in areas with limited resources? | Women aged 65 years with evidence of one adequate negative prior screening test result and no history of CIN 2 or higher | Women aged 65 years with evidence of two adequate negative prior screening test results and no history of CIN 2 or higher | Women with a total hysterectomy and no history of CIN 2 or higher | Sexually naive | Any of the above | – | – | – |
|  | 7.4% | 59.3% | 4.9% | 3.7% | 24.7% | – | – | – |
| What is the optimal strategy for people with abnormal cytology suggesting a premalignant lesion or other abnormal screening results in areas with limited resources? | Colposcopy referral for a positive test, followed by biopsy and treatment only if CIN 2 or higher confirmed on biopsy result | Colposcopy referral for a positive test, followed by biopsy | Proceed to LEEP/conization (LLETZ, cryotherapy, or thermal coagulation) | Repeat the cervical smear and colposcopy-guided biopsies | Abstain | – | – | – |
|  | 52.6% | 24.1% | 19.6% | 3.8% | 0% | – | – | – |
| What is the optimal strategy for people with premalignant lesion in a cervical biopsy in areas with limited resources? | Treatment only if CIN 2 or higer confirmed on biopsy result | Treatment only if CIN 3 confirmed on biopsy result | Proceed to LEEP/conization (LLETZ, cryotherapy, or thermal coagulation) | Repeat the cervical smear and colposcopy-guided biopsies in 6 months | Abstain | – | – | – |
|  | 48.0% | 13.8% | 35.8% | 2.4% | 0% | – | – | – |
| What is the best periodicity of cervical cancer screening (pap smear) in an immunocompromised population in areas with limited resources? | Every 6 months | Every year | Every 2 years | Every 3 years | Every 6 months; then every year after 2 normal exams | Every year; then every two years after 2 normal exams | None | Abstain |
|  | 13.5% | 35.3% | 1.5% | 5.3% | 32.3% | 12.0% | 0% | 0% |

Answers to not all questions may total 100% due to rounding.

CIN, cervical intraepithelial neoplasia; LEEP, loop electrosurgical excision procedure; LLETZ, large loop excision of the transformation zone.

**Supplementary table 4: Questions related to the diagnosis of cervical cancer**

| **Question** | **Answers and frequency of responses** | | | | |
| --- | --- | --- | --- | --- | --- |
| When is immunohistochemistry indicated in cervical cancer in areas with limited resources? | Always | Never | In adenocarcinoma | In suspected adenocarcinoma, neuroendocrine carcinoma, sarcoma, or rare tumors | Abstain |
|  | 11.0% | 13.6% | 1.7% | 72.0% | 1.7% |
| Is cervical cytology (positive for carcinoma) sufficient for diagnosis of clinically suspicious tumor in areas with limited resources? | Yes | No | Only if suspicious for or positive for high-grade intraepithelial lesions or carcinoma/malignancy | Abstain | – |
|  | 17.7% | 59.3% | 21.2% | 1.8% | – |
| Is colposcopy indicated for cervical cancer diagnosis in areas with limited resources? | Always | Never | When cytology is positive | Abstain | – |
|  | 13.4% | 9.0% | 76.9% | 0.8% | – |
| Which minimal information should be listed in a histopathological report for resected surgical specimens in areas with limited resources? | Margin, tumor size, and tumor grade | Margin, tumor size, tumor grade, and depth of invasion | Margin, tumor size, tumor grade, depth of invasion, lymphovascular invasion, mitotic index, necrosis, perineural invasion, parametrium involvement, and lymph-node metastasis | None | Abstain |
|  | 1.4% | 16.4% | 80.0% | 2.1% | 0% |

Answers to not all questions may total 100% due to rounding.

**Supplementary table 5: Questions related to staging of cervical cancer (stages refer to the latest International Federation of Obstetrics and Gynecology classification^(11)^).**

| **Question** | **Answers and frequency of responses** | | | | | |
| --- | --- | --- | --- | --- | --- | --- |
| What are the diagnostic staging tools for patients with signs and symptoms of cervical cancer in areas with limited resources? | Immediate outpatient biopsy, CT scan, blood count, renal and liver tests, and chest X-ray | Immediate outpatient biopsy, abdominal and pelvic ultrasound , blood count, renal and liver tests, and chest X-ray | Immediate outpatient biopsy, abdominal-pelvic MRI, blood count, renal and liver tests, and chest X-ray | Abstain | – | – |
|  | 29.1% | 43.6% | 24.8% | 2.6% | – | – |
| After microscopic cervical cancer is diagnosed in a cone specimen (stage IA1 with positive lymphovascular involvement or IA2), is it necessary to perform any additional staging exam in areas with limited resources? | Abdominal/pelvic/transvaginal ultrasound and chest X-ray | Abdominal and pelvic CT and chest X-ray | None | Abstain | – | – |
|  | 20.7% | 48.3% | 30.3% | 0.7% | – | – |
| Which additional imaging method is indicated for clinical stage 1B1 or smaller cervical cancer in areas with limited resources? | Abdominal/pelvic/transvaginal ultrasound and chest X-ray | Abdominal and pelvic CT and chest X-ray | None | Abstain | – | – |
|  | 27.9% | 62.1% | 9.3% | 0.7% | – | – |
| Which additional imaging method is indicated for clinical 1B1 stage or smaller cervical cancer if radical trachelectomy is being considered in areas with limited resources? | Abdominal/pelvic ultrasound and chest X-ray | Abdominal and pelvic CT and chest X-ray | Abdominal and pelvic MRI and chest X-ray | None | Abstain | – |
|  | 6.4% | 32.6% | 57.5% | 2.1% | 1.4% | – |
| Which additional imaging method is indicated for clinical IB2-3 stage in areas with limited resources? | Abdominal and pelvic CT and chest X-ray | Abdominal/pelvic ultrasound and chest X-ray | None | Abstain | – | – |
|  | 80.0% | 15.6% | 3.0% | 1.5% | – | – |
| Which additional imaging method is indicated for clinical II-IVA stages in areas with limited resources? | Abdominal and pelvic CT and chest X-ray | Abdominal/pelvic ultrasound and chest X-ray | None | Abstain | – | – |
|  | 85.2% | 12.0% | 2.1% | 0.7% | – | – |
| Which is the next step in patients with suspicious pelvic and/or para-aortic lymph nodes in imaging studies (ultrasound or CT) areas with limited resources? | Surgical staging | Image-guided transparietal biopsy | Abdominal and pelvic CT | Start treatment without further investigation | None | Abstain |
|  | 27.5% | 13.4% | 19.7% | 38.7% | 0% | 0.7% |

Answers to not all questions may total 100% due to rounding.

CT, computed tomography; MRI, magnetic resonance imaging.

**Supplementary table 6.** Questions related to surveillance of cervical cancer.

| **Question** | **Answers and frequency of responses** | | | | | | |
| --- | --- | --- | --- | --- | --- | --- | --- |
| How often do you follow up patients treated with early stage disease after curative treatment in areas with limited resources? | Every 3 months in the first 2 years, after that, every 6 months until 5 years from the treatment | Every 6 months until 5 years from the treatment | Annually until 5 years from the treatment | Every 6 months in the first 2 years, after that, annually until 5 years from the treatment | None | Abstain | – |
|  | 58.4% | 13.6% | 0.8% | 26.4% | 0% | 0.8% | – |
| For early-stage cervical cancer patients who were submitted to a radical hysterectomy, should vaginal cytology be performed in areas with limited resources? | No | Yes | Abstain | – | – | – | – |
|  | 38.4% | 60.8% | 0.8% | – | – | – | – |
| What is the best follow-up assessment for early stage cervical cancer patients who have had curative treatment in areas with limited resources? | Physical exam | Physical exam and lab tests | Physical exam, lab tests and vaginal cytology | Physical exam, vaginal cytology, abdominal-pelvic CT, and chest X-ray | Physical exam, vaginal cytology, HPV-DNA, abdominal-pelvic US, and chest X-ray | None | Abstain |
|  | 36.4% | 3.9% | 39.5% | 14.0% | 3.1% | 2.3% | 0.8% |
| How often do you follow up patients treated with locally advanced disease after curative treatment in areas with limited resources? | Every 3 months in the first 2 years, after that, every 6 months until 5 years from the treatment | Every 6 months until 5 years from the treatment | Annually until 5 years from the treatment | Every 6 months in the first 2 years, after that, annually until 5 years from the treatment | None | Abstain | – |
|  | 80.5% | 4.7% | 1.6% | 11.7% | 1.6% | 0% | – |
| What is the recommended follow-up assessment for locally advanced stage cervical cancer patients who have had curative treatment in areas with limited resources? | Physical exam | Physical exam, HPV DNA and lab working | Physical exam, lab workup, and vaginal cytology | Physical exam, vaginal cytology, abdominal-pelvic CT, and chest X-ray | Physical exam, abdominal-pelvic CT, and chest X-ray | None | Abstain |
|  | 21.9% | 2.3% | 25.0% | 34.4% | 16.4% | 0% | 0% |

Answers to not all questions may total 100% due to rounding.

CT, computed tomography.

**Supplementary Table 7: Treatment of early-stage cervical cancer**

| **Question** | **Answers (%)** | | | | | | | |
| --- | --- | --- | --- | --- | --- | --- | --- | --- |
| What is your treatment recommendation for women with stage IA2 cervical cancer in areas of limited resources (no fertility desired)? | Chemoradiation alone | Radiation alone | Radical hysterectomy | Simple hysterectomy | Radical Trachelectomy | Conization | Abstain | - |
|  | 2.2% | 3.0% | 65.7% | 23.1% | 3.7% | 0% | 2.2% | - |
| What is your treatment recommendation for women with stage IA2 cervical cancer in areas where surgeons do not have a full training in gynecology oncology (no fertility desired)? | Chemoradiation alone | Radiation alone | Simple hysterectomy | Radical hysterectomy | Conization | Abstain | - | - |
|  | 19.2% | 15.1% | 45.2% | 12.3% | 0.7% | 7.5% | - | - |
| What is your treatment recommendation for women with stage IA2 cervical cancer in areas of limited resources (fertility desired)? | Chemoradiation alone | Radiation alone | Radical hysterectomy | Simple hysterectomy | Trachelectomy | Conization | Abstain | - |
|  | 0% | 0% | 1.4% | 0% | 77.6% | 18.2% | 2.8% | - |
| What is your treatment recommendation for women with stage IA2 cervical cancer in areas where surgeons do not have a full training in gynecology oncology (fertility desired)? | Chemoradiation alone | Radiation alone | Simple hysterectomy | Conization | Abstain | - | - | - |
|  | 4.9% | 7.3% | 3.3% | 67.5% | 17.1% | - | - | - |
| What is your treatment recommendation for women with stage IB1-IB2 cervical cancer in areas of limited resources? | Surgery alone | Radiation alone | Chemoradiation | Chemoradiation  **followed by surgery** | Surgery followed by radiation +/- chemotherapy | Neoadjuvant chemotherapy followed by surgery | Abstain | - |
|  | 40.8% | 1.6% | 16.0% | 4.8% | 30.4% | 5.6% | 0.8% | - |
| What is your treatment recommendation for women with stage IB1-IB2 cervical cancer when radiotherapy is not available? | Surgery alone | Surgery followed by chemotherapy | Neoadjuvant chemotherapy followed by surgery | Chemotherapy alone | Abstain | - | - | - |
|  | 63.4% | 18.7% | 14.9% | 0.8% | 2.2% | - | - | - |
| What is your treatment recommendation for women with stage IB1-IB2 cervical cancer in areas where surgeons do not have a full training in gynecology oncology? | Surgery alone | Radiation alone | Chemoradiation | Chemoradiation followed by surgery | Surgery followed by radiation +/- chemotherapy | Neoadjuvant chemotherapy followed by surgery | Abstain | - |
|  | 3.7% | 14.9% | 61.2% | 1.5% | 10.5% | 6.0% | 2.2% | - |
| What is your treatment recommendation for women with stage IB1-IIA cervical cancer when radiotherapy is not available and surgeons do not have a full training in gynecology oncology? | Surgery alone | Surgery followed by chemotherapy | Neoadjuvant chemotherapy followed by surgery | Chemotherapy alone | Abstain | - | - | - |
|  | 13.9% | 15.6% | 41.0% | 11.5% | 18.0% | - | - | - |
| What is your treatment recommendation for women with cancer confined to the cervix with a clinically visible tumor >4 cm (stage IB3) to IIA in areas of limited resources? | Chemoradiation alone | Chemoradiation followed by surgery | Surgery followed by chemoradiation | Neoadjuvant chemotherapy followed by surgery | Neoadjuvant chemotherapy followed by surgery and radiotherapy | Neoadjuvant chemotherapy followed by radiotherapy | Abstain | - |
|  | 70.0% | 6.4% | 5.0% | 10.7% | 5.7% | 2.1% | 0% | - |
| What is your treatment recommendation for women with cancer confined to the cervix with a clinically visible tumor >4 cm (stage IB3) to IIA in areas when radiotherapy is not available? | Primary surgery alone | Surgery followed by chemotherapy | Neoadjuvant chemotherapy followed by surgery | Abstain | - | - | - | - |
|  | 11.8% | 10.1% | 75.6% | 2.5% | - | - | - | - |
| What is your treatment recommendation for women with cancer confined to the cervix with a clinically visible tumor >4 cm (stage IB3) to IIA in areas where surgeons do not have a full training in gynecology oncology? | Chemoradiation alone | Neoadjuvant chemotherapy followed by simple hysterectomy | Neoadjuvant chemotherapy followed by simple hysterectomy and radiotherapy | Neoadjuvant chemotherapy followed by radiotherapy | Abstain | - | - | - |
|  | 81.4% | 3.1% | 7.0% | 8.5% | 0% | - | - | - |
| What is your treatment recommendation for women with cancer confined to the cervix with a clinically visible tumor >4 cm (stage IB3) to IIA in areas when radiotherapy is not available and surgeons do not have a full training in gynecology oncology? | Simple hysterectomy alone | Simple hysterectomy followed by chemotherapy | Neoadjuvant chemotherapy followed by simple hysterectomy | Abstain | - | - | - | - |
|  | 4.2% | 0.8% | 75.8% | 19.2% | - | - | - | - |
| After an incidental diagnosis of IA2 without lymphovascular invasion in simple hysterectomy specimen, and absence of enlarged pelvic lymph nodes evaluated by CT scan, what is the best course of action in an area without qualified surgeons in gynecologic oncology? | Strict follow-up | External radiotherapy | Concurrent chemoradiation | Pelvic lymphadenectomy | Abstain | - | - | - |
|  | 69.7% | 10.7% | 10.7% | 6.6% | 2.5% | - | - | - |
| After a diagnosis of IA1 cervical cancer with positive lymphovascular space involvement in LEEP specimen without pregnancy desire, what is the best course of action in an area without qualified surgeons in gynecologic oncology? | Strictly follow-up | Simple hysterectomy | Concurrent chemoradiation | Radiation alone | Simple hysterectomy and pelvic lymphadenectomy | Abstain | - | - |
|  | 14.4% | 43.3% | 11.5% | 5.8% | 23.1% | 1.9% | - | - |
| What is the recommended approach for radical hysterectomy in a patient with stage IB-IIA cervical cancer in an area with limited resources? | Open radical hysterectomy | Laparoscopic assisted vaginal radical hysterectomy | Laparoscopic radical hysterectomy | Any minimally invasive approach | Abstain | - | - | - |
|  | 95.2% | 0.8% | 2.4% | 0% | 1.6% | - | - | - |
| What is the recommended treatment for a stage IB1-IIA cervical cancer patient with poor clinical performance in an area with limited resources? | Radical hysterectomy | Simple hysterectomy | Neoadjuvant chemotherapy followed by radical hysterectomy | Neoadjuvant chemotherapy followed by simple hysterectomy | Radiotherapy | Chemoradiation | Abstain | - |
|  | 17.9% | 1.9% | 2.8% | 2.8% | 32.1% | 41.5% | 0.9% | - |
| What is your treatment recommendation for women with early stages of cervix cancer after surgery with at least two intermediate-risk features (lymphovascular invasion, cervical stromal invasion >10mm, or tumor size ≥4 cm) in areas with limited resources? | Observation | Adjuvant radiotherapy alone | Adjuvant radiotherapy and chemotherapy | Chemotherapy alone | Abstain | - | - | - |
|  | 6.2% | 48.5% | 43.3% | 2.1% | 0% | - | - | - |
| What is your treatment recommendation for women with early stages of cervix cancer after surgery with at least one high-risk features (positive surgical margins, pathologically involved pelvic nodes, or positive involvement of the parametria) in areas with limited resources? | Observation | Adjuvant radiotherapy alone | Adjuvant radiotherapy and chemotherapy | Chemotherapy alone | Abstain | - | - | - |
|  | 0.8% | 14.6% | 80.0% | 4.6% | 0% | - | - | - |
| What external radiotherapy technique is recommended as the minimum required treatment for women with early-stage cervix cancer who need adjuvant radiotherapy after surgery in areas with limited resources? | Conventional (2D) | Conformal | Abstain | - | - | - | - | - |
|  | 64.0% | 32.0% | 4.0% | - | - | - | - | - |
| In Institutions where there is only conventional radiotherapy technique, patients with early stages of cervical cancer can be treated with primary or adjuvant external radiotherapy? | Yes | No | Abstain | - | - | - | - | - |
|  | 94.0% | 4.0% | 2.0% | - | - | - | - | - |
| In Institutions where there is only cobalt machine, patients with early stages of cervical cancer can be treated with external radiotherapy? | Yes | No | Abstain | - | - | - | - | - |
|  | 72.5% | 22.5% | 5.0% | - | - | - | - | - |
| Do you recommend adjuvant vaginal vault brachytherapy alone instead of external radiotherapy for patients with early-stage cervical cancer and at least two intermediate risk features (lymphovascular invasion, cervical stromal invasion, or tumor size ≥4 cm)? | Yes | No | Abstain | - | - | - | - | - |
|  | 33.3% | 64.9% | 1.8% | 0% | - | - | - | - |
| Do you recommend vaginal vault brachytherapy after external radiotherapy, as boost, for patients with early stage cervical cancer and at least two intermediate risk features (lymphovascular invasion, cervical stromal invasion, or tumor size ≥4 cm)? | Yes | No | Abstain | - | - | - | - | - |
|  | 65.0% | 33.3% | 1.7% | - | - | - | - | - |
| Do you recommend vaginal vault brachytherapy after external radiotherapy, as boost, for patients with early stage cervical cancer and at least one high-risk feature (positive surgical margins, pathologically involved pelvic nodes, or positive involvement of the parametria)? | Yes, always | Yes, but only for patients with positive vaginal margins | No | Abstain | - | - | - | - |
|  | 59.0% | 34.4% | 6.6% | 0% | - | - | - | - |
| In patients with cervical cancer scheduled for radical hysterectomy and pelvic lymphadenectomy, if you find a suspicious lymph node in the beginning of the surgery, what is the recommended assessment in an area of limited resources? | Proceed with surgery as planned | Resect the suspicious lymph node and send it to frozen section. In case of a confirmed metastasis, abort the surgery without any further dissection. | Resect the suspicious lymph node and send it to frozen section. In case of a confirmed metastasis, perform bilateral pelvic lymphadenectomy and keep the uterus. | Resect the suspicious lymph node and send it to frozen section. In case of a confirmed metastasis, perform bilateral pelvic and para-aortic lymphadenectomies and keep the uterus. | Abort surgery and proceed with chemoradiation | Abstain | - | - |
|  | 17.3% | 38.2% | 6.4% | 19.1% | 18.2% | 0.9% | - | - |
| Regarding an area without access to radiotherapy: in patents with cervical cancer scheduled for radical hysterectomy and pelvic lymphadenectomy, if you find a suspicious lymph node at the beginning of the surgery, what is the best management? | Proceed with surgery as planned | Resect the suspicious lymph node and send it to frozen section. In case of a confirmed metastasis, abort the surgery without any further dissection. | Resect the suspicious lymph node and send it to frozen section. In case of a confirmed metastasis, perform bilateral pelvic lymphadenectomy and keep the uterus. | Resect the suspicious lymph node and send it to frozen section. In case of a confirmed metastasis, perform bilateral pelvic and para-aortic lymphadenectomies and keep the uterus. | Resect the suspicious lymph node and send it to frozen section. In case of a confirmed metastasis, perform bilateral pelvic and para-para-aortic lymphadenectomies, and perform the radical hysterectomy. | Abort surgery and proceed with chemotherapy | Abstain | - |
|  | 44.1% | 6.3% | 1.8% | 2.7% | 42.3% | 0.9% | 1.8% | - |
| What is the maximum tumor size you consider appropriate to perform conization as fertility sparing surgery in an area without adequate surgeons (FIGO 2018)? | Up to IA1 | Up to IA2 | Up to IB1 | Up to IB2 | I do not recommend in this scenario | Abstain | - | - |
|  | 40.7% | 34.5% | 18.6% | 2.7% | 2.7% | 0.9% | - | - |
| What is the maximum tumor size you consider appropriate to perform radical trachelectomy as fertility sparing surgery in an area without access to adequate surgeons (FIGO 2018)? | Up to IA1 | Up to IA2 | Up to IB1 | Up to IB2 | I do not recommend in this scenario | Abstain | - | - |
|  | 9.6% | 20.0% | 20.8% | 1.6% | 45.6% | 2.4% | - | - |
| Should radical trachelectomy be proposed in a patient with stage IB1 cervical cancer in an area without access to adequate surgeons? | Yes, and the gynecologist should try to perform the radical trachelectomy | Yes, but the patient should be referred to another Service | Yes, but the patient should be submitted to neoadjuvant chemotherapy | No | Abstain | - | - | - |
|  | 5.0% | 49.6% | 3.4% | 41.2% | 0.8% | - | - | - |

**Supplementary Table 8: Treatment of locally advanced-stage cervical cancer**

| **Questions** | **Answers (%)** | | | | | | | |
| --- | --- | --- | --- | --- | --- | --- | --- | --- |
| In areas with limited resources, what is your treatment recommendation for women with stages IIB through IIIA cervical cancer? | Surgery followed by radiotherapy | Surgery followed by chemoradiation | Neoadjuvant chemotherapy followed by surgery | Primary concomitant chemoradiation | Neoadjuvant chemotherapy followed by surgery and radiotherapy | Neoadjuvant chemotherapy followed by radiotherapy | Abstain | - |
|  | 0% | 5.2% | 3.5% | 86.1% | 3.5% | 1.7% | 0% | - |
| In areas with limited resources, what is your treatment recommendation for women with stages IIIB, IIIC and IVA cervical cancer? | Radiation therapy alone | Primary concomitant chemoradiation | Neoadjuvant chemotherapy followed radiotherapy | Chemotherapy alone | Abstain | - | - | - |
|  | 0.9% | 90.4% | 4.4% | 4.4% | 0% | - | - | - |
| In patients with locally advanced cervical cancer, what is the best treatment in an area with limited resources, where chemotherapy is not available in a timely manner (at least 8-12 weeks to be started)? | Radiotherapy alone | Radiotherapy followed by surgery | Surgery alone | Surgery followed by radiation | None | Abstain | - | - |
|  | 72.1% | 11.5% | 1.0% | 9.6% | 1.9% | 3.9% | - | - |
| In patients with locally advanced cervical cancer, what is the best treatment in an area with limited resources, when radiotherapy is not available? | Chemotherapy alone | Neoadjuvant chemotherapy followed by surgery | Surgery alone | Surgery followed by chemotherapy | None | Abstain | - | - |
|  | 12.5% | 71.4% | 0.9% | 8.0% | 0.9% | 6.3% | - | - |
| In patients with locally advanced cervical cancer, what is the best treatment in an area with limited resources when surgeons do not have a full training in gynecology oncology? | Chemoradiation alone | Chemoradiation followed by surgery | Surgery followed by chemoradiation | Neoadjuvant chemotherapy followed by surgery | Neoadjuvant chemotherapy followed by surgery and radiotherapy | Neoadjuvant chemotherapy followed by radiotherapy | Abstain | - |
|  | 86.5% | 2.9% | 1.9% | 1.9% | 1.0% | 5.8% | 0% | - |
| In areas with limited resources, which external radiotherapy technique is recommended as a minimal option for women with stages IB3 through IVA cervical cancer with or without concomitant chemotherapy? | Conformal | Conventional | Abstain | - | - | - | - | - |
|  | 35.9% | 64.1% | 0% | - | - | - | - | - |
| In Institutions where there is only conventional radiotherapy technique, can patients with stages IB3 through IVA be treated with external radiotherapy? | Yes, for all stages | Yes, only for stage IIB through IVA | No | Abstain | - | - | - | - |
|  | 69.3% | 24.8% | 4.0% | 2.0% | - | - | - | - |
| In Institutions where there is only cobalt machine, can patients with stages IB3 through IVA cervical cancer be treated with external radiotherapy? | Yes, for all stages | Yes, only for stage IIB through IVA | No | Abstain | - | - | - | - |
|  | 70.7% | 22.0% | 6.1% | 1.2% | - | - | - | - |
| Which brachytherapy technique is recommended as the minimal option for patient with stages IB3 through IVA after external radiation and eligible for applicators placement in areas with limited resources? | Three-dimensional with CT or MRI based planning known as Imaged Guided Adaptative Brachytherapy (IGABT) | Two-dimensional convention technique | Abstain | - | - | - | - | - |
|  | 17.1% | 80.5% | 2.4% | - | - | - | - | - |
| In areas with limited resources where no brachytherapy is available, how do you treat patients with cervical cancer stages IB3 through IIA? | Surgery | External-beam radiotherapy alone | External-beam radiotherapy with chemotherapy | External-beam radiotherapy followed by surgery if residual disease | Neoadjuvant chemotherapy followed by surgery | Neoadjuvant chemoradiation followed by surgery | Neoadjuvant chemotherapy followed by surgery and radiation | Abstain |
|  | 5.1% | 4.0% | 49.5% | 13.1% | 5.1% | 14.1% | 5.1% | 4.0% |
| In areas with limited resources where no brachytherapy is available, how do you treat patients with cervical cancer stages IIB through IVA? | Surgery | External-beam radiotherapy alone | External-beam radiotherapy with chemotherapy | External-beam radiotherapy followed by surgery if residual disease | Neoadjuvant chemotherapy followed by surgery | Neoadjuvant chemoradiation followed by surgery | Neoadjuvant chemotherapy followed by surgery and radiation | Abstain |
|  | 0% | 2.0% | 67.0% | 13.0% | 1.0% | 10.0% | 6.0% | 1.0% |
| For women with stages IB3 through IVA treated by primary chemoradiation or radiotherapy alone, which is the maximal acceptable duration of radiotherapy (whole pelvic irradiation + brachytherapy or external-beam boost) in areas with limited resources? | 7 weeks | 12 weeks | 15 weeks | 20 weeks | Abstain | - | - | - |
|  | 50.7% | 36.0% | 4.0% | 4.0% | 5.3% | - | - | - |
| For women with cervical cancer and suspected or pathologically confirmed para-aortic node involvement, what is your treatment recommendation in areas with limited resources? | Primary chemoradiation with extended-field radiotherapy | Chemotherapy alone | Radiation alone | Neoadjuvant chemotherapy followed by radiotherapy | Chemoradiation followed by surgery | Surgery followed by radiation +/- chemotherapy | Abstain | - |
|  | 86.1% | 0% | 0% | 5.0% | 1.0% | 6.9% | 1.0% | - |
| In patients with locally advanced cervical cancer and poor geriatric score and/or poor performance status, what is the best treatment? | Chemotherapy | Chemoradiation | Chemoradiation followed by chemotherapy | Chemotherapy followed by chemoradiation | Radiation | Surgery | Best supportive care | Abstain |
|  | 3.6% | 30.9% | 2.7% | 0.9% | 50.9% | 0.9% | 10.0% | 0.0% |
| In patient with locally advanced cervical cancer and poor geriatric score and/or poor performance status, what is the best treatment, if radiotherapy is not available in an area with limited resources? | Chemotherapy alone | Neoadjuvant chemotherapy followed by surgery if resectable | Surgery alone if resectable | Surgery, if resectable, followed by chemotherapy | Best supportive care | Abstain | - | - |
|  | 21.2% | 8.5% | 5.1% | 1.7% | 59.3% | 4.2% | - | - |
| What is the best treatment for HIV/AIDS and other immunosuppressed patients with locally advanced cervical cancer in an area with limited resources? | Chemotherapy | Chemoradiation | Chemoradiation followed by chemotherapy | Chemotherapy followed by chemoradiation | Radiation alone | Surgery if resectable | Surgery followed by chemoradiation or radiation | Abstain |
|  | 2.9% | 79.6% | 4.9% | 0% | 7.8% | 1.9% | 2.9% | 0% |
| In patient with locally advanced cervical cancer, what is the preferred radiosensitizing agent in an area with limited resources? | Weekly cisplatin | Cisplatin every 3 weeks | Cisplatin and fluorouracil | Carboplatin | Fluorouracil | Cisplatin and gemcitabine | Gemcitabine | Abstain |
|  | 78.7% | 15.7% | 0% | 3.4% | 0% | 1.1% | 0% | 1.1% |
| In patients with locally advanced cervical cancer and not eligible to receive to cisplatin, what is the best radiosensitizing agent in an area with limited resources? | Carboplatin | Carboplatin and fluorouracil | Fluorouracil | Taxane | Gemcitabine | None | Abstain | - |
|  | 73.8% | 7.1% | 7.1% | 3.6% | 3.6% | 4.8% | 0% | - |
| In AIDS and other immunosuppressed patients with locally advanced cervical cancer, what is the best radiosensitizing agent in an area with limited resources? | Weekly cisplatin | Cisplatin every 3 weeks | Gemcitabine | Carboplatin | Fluorouracil | Taxane | None | Abstain |
|  | 94.3% | 3.5% | 1.2% | 1.2% | 0% | 0% | 0% | 0% |
| If you recommend neoadjuvant chemotherapy in locally advanced cervical cancer, what is the best chemotherapy regimen in an area with limited resources? | Carboplatin and paclitaxel | Cisplatin and paclitaxel | Carboplatin and gemcitabine | Cisplatin and gemcitabine | Cisplatin and fluorouracil | Paclitaxel, ifosfamide and cisplatin | Cisplatin alone | Abstain, including I do not recommend neoadjuvant chemotherapy |
|  | 49.4% | 25.3% | 0% | 2.3% | 3.5% | 1.2% | 3.5% | 14.9% |
| If you recommend consolidation chemotherapy after chemoradiation in locally advanced cervical cancer, what is the best chemotherapy regimen in an area with limited resources? | Cisplatin and gemcitabine | Carboplatin and gemcitabine | Cisplatin and fluorouracil | Carboplatin and paclitaxel | Platinum alone | Gencitabine | Fluorouracil | Abstain, including I do not recommend consolidation chemotherapy after chemoradiation |
|  | 18.0% | 2.6% | 2.6% | 28.2% | 6.4% | 3.9% | 0.0% | 38.5% |
| In patients with locally advanced cervical cancer that undergo chemoradiation and persist with only residual disease in the cervix, which surgery is indicated, in an area with limited resources? | Simple hysterectomy and BSO | Radical hysterectomy (and BSO) | Pelvic exenteration | Any surgery that provides free margins | I do not recommend surgery | Abstain | - | - |
|  | 36.5% | 23.5% | 8.2% | 22.4% | 8.2% | 1.2% | - | - |
| Should ovarian transposition in locally advanced cervical cancer setting, be always offering in a reproductive age to women in an area with limited resources? | Never | Yes, if there is no ovarian involvement | Yes, if squamous cell histology | Alternatives 2 and 3 | Abstain | - | - | - |
|  | 26.8% | 28.9% | 5.2% | 35.1% | 4.1% | - | - | - |
| In locally advanced cervical cancer, with bulky tumors (>4 cm) and no residual tumor after treatment, when do you indicate hysterectomy after chemoradiation in an area with limited resources? | Never | If brachytherapy is not available | In adenocarcinoma histology | Alternatives 2 and 3 | Abstain | - | - | - |
|  | 66.3% | 16.3% | 2.2% | 13.0% | 2.2% | - | - | - |

**Supplementary Table 9: Treatment of advanced-stage cervical cancer**

| **Question** | **Answers and frequency of responses** | | | | | | | |
| --- | --- | --- | --- | --- | --- | --- | --- | --- |
| What is the recommended first-line treatment for patients with platinum-naive metastatic or recurrent cervical cancer not amenable to salvage loco-regional treatment when all resources are available? | Cisplatin, 50 mg/m² IV on day 1 with Paclitaxel, 175 mg/m² IV on day 1 and Bevacizumab, 15 mg/kg IV on day 1 every 3 weeks | Cisplatin, 50 mg/m² IV on day 1 with Paclitaxel, 175 mg/m² IV on day 1 every 3 weeks | Carboplatin AUC 5 IV on day 1 with Paclitaxel, 175 mg/m² IV on day 1 and Bevacizumab, 15 mg/kg IV on day 1 every 3 weeks | Carboplatin AUC 5 IV on day 1 with Paclitaxel, 175 mg/m² IV on day 1 every 3 weeks | Cisplatin, 50 mg/m² IV on day 1 in association with 5-FU, 1000 mg/m² IV on days 1-4 every 3 weeks | Cisplatin, 50 mg/m² IV on day 1 | Best supportive care | Abstain |
|  | 69.2% | 3.9% | 23.1% | 1.9% | 0% | 1.9% | 0% | 0% |
| What is the recommended first-line treatment for patients with platinum-naive metastatic or recurrent cervical cancer not amenable to salvage loco-regional treatment eligible to cisplatin in an area with limited resources? | Cisplatin, 50 mg/m² IV on day 1 with Paclitaxel, 175 mg/m² IV on day 1 every 3 weeks | Carboplatin AUC 5 IV on day 1 with Paclitaxel, 175 mg/m² IV on day 1 every 3 weeks | Cisplatin, 50 mg/m² IV on day 1 in association with 5-FU, 1000 mg/m² IV on days 1-4 every 3 weeks | Other platinum-based chemotherapy doublet | Monotherapy with platinum agent | Best supportive care | Abstain | - |
|  | 60.7% | 26.8% | 8.9% | 1.8% | 1.8% | 0% | 0% | - |
| What is the recommended treatment for patients with prior platinum (> 6 months) metastatic or recurrent cervical cancer not amenable to salvage loco-regional treatment when all resources are available? | Cisplatin, 50 mg/m² IV on day 1 with Paclitaxel, 175 mg/m² IV on day 1 and Bevacizumab, 15 mg/kg IV on day 1 every 3 weeks | Cisplatin, 50 mg/m² IV on day 1 with Paclitaxel, 175 mg/m² IV on day 1 every 3 weeks | Carboplatin AUC 5 IV on day 1 with Paclitaxel, 175 mg/m² IV on day 1 and Bevacizumab, 15 mg/kg IV on day 1 every 3 weeks | Carboplatin AUC 5 IV on day 1 with Paclitaxel, 175 mg/m² IV on day 1 every 3 weeks | Other platinum-based chemotherapy doublet | Monotherapy with platinum agent | Best supportive care | Abstain |
|  | 53.7% | 1.5% | 37.3% | 3.0% | 0% | 0% | 0% | 4.5% |
| What is the recommended treatment for patients with prior platinum (>6 months) metastatic or recurrent cervical cancer not amenable to salvage loco-regional treatment in an area with limited resources? | Cisplatin, 50 mg/m² IV on day 1 with Paclitaxel, 175 mg/m² IV on day 1 every 3 weeks | Carboplatin AUC 5 IV on day 1 with Paclitaxel, 175 mg/m² IV on day 1 every 3 weeks | Other platinum-based chemotherapy doublet | Monotherapy with platinum agent | Monotherapy with non-platinum agent | Best supportive care | Abstain | - |
|  | 57.4% | 37.7% | 1.6% | 1.6% | 0% | 1.6% | 0% | - |
| What is the recommended treatment for patients with prior platinum (< 6 months) metastatic or recurrent cervical cancer not amenable to salvage loco-regional treatment when all resources are available? | Cisplatin, 50 mg/m² IV on day 1 with Paclitaxel, 175 mg/m² IV on day 1 and Bevacizumab, 15 mg/kg IV on day 1 every 3 weeks | Cisplatin, 50 mg/m² IV on day 1 with Paclitaxel, 175 mg/m² IV on day 1 every 3 weeks | Carboplatin AUC 5 IV on day 1 with Paclitaxel, 175 mg/m² IV on day 1 and Bevacizumab, 15 mg/kg IV on day 1 every 3 weeks | Carboplatin AUC 5 IV on day 1 with Paclitaxel, 175 mg/m² IV on day 1 every 3 weeks | Other platinum-based chemotherapy doublet | Monotherapy with platinum agent | Best supportive care | Abstain |
|  | 27.3% | 3.6% | 49.1% | 3.6% | 0% | 1.8% | 7.3% | 7.3% |
| What is the recommended treatment for patients with prior platinum  (< 6 months) metastatic or recurrent cervical cancer not amenable to salvage loco-regional treatment in area with limited resources? | Cisplatin, 50 mg/m² IV on day 1 with Paclitaxel, 175 mg/m² IV on day 1 every 3 weeks | Carboplatin AUC 5 IV on day 1 with Paclitaxel, 175 mg/m² IV on day 1 every 3 weeks | Other platinum-based chemotherapy doublet | Monotherapy with platinum agent | Monotherapy with non-platinum agent | Best supportive care | Abstain | - |
|  | 13.5% | 51.9% | 1.9% | 1.9% | 25.0% | 5.8% | 0% | - |
| What is the recommended first-line treatment for patients with metastatic or recurrent cervical cancer not amenable to salvage loco-regional treatment not eligible to receive cisplatin when all resources are available? | Topotecan, 0.75 mg/m² IV on day 1 to 3 with Paclitaxel, 175 mg/m² IV on day 1 and Bevacizumab, 15 mg/kg IV on day 1 every 3 weeks | Carboplatin AUC 5 IV on day 1 with Paclitaxel, 175 mg/m² IV on day 1 and Bevacizumab, 15 mg/kg IV on day 1 every 3 weeks | Carboplatin AUC 5 IV on day 1 with Paclitaxel, 175 mg/m² IV on day 1 every 3 weeks | Carboplatin AUC 5 IV with Gemcitabine, 1000 mg/m² IV on days 1,8 every 3 weeks | Carboplatin AUC 5 IV every 3 weeks | Monotherapy with non-platinum agent | Best supportive care | Abstain |
|  | 30.9% | 67.3% | 0% | 0% | 0% | 0% | 1.8% | 0% |
| What is the recommended first-line treatment for patients with metastatic or recurrent cervical cancer not amenable to salvage loco-regional treatment not eligible to receive cisplatin in an area with limited resources? | Carboplatin AUC 5 IV on day 1 with Paclitaxel, 175 mg/m² IV on day 1 every 3 weeks | Carboplatin AUC 5 IV with Gemcitabine, 1000 mg/m² IV on days 1,8 every 3 weeks | Carboplatin AUC 5 IV with 5FU 1000 mg/m² IV on days 1-4 every 3 weeks | Carboplatin AUC 5 IV every 3 weeks | Monotherapy with non-platinum agent | Best supportive care | Abstain | - |
|  | 76.1% | 6.5% | 0% | 4.4% | 8.7% | 2.2% | 2.2% | - |
| What is the recommended first-line treatment for AIDS and other immunosuppressed patients with metastatic or recurrent cervical cancer not amenable to salvage loco-regional treatment in an area with limited resources? | Full-dose platinum-based chemotherapy doublet | Adjusted-dose platinum-based chemotherapy doublet | Monotherapy with platinum agent | Monotherapy with non-platinum agent | Best supportive care | Abstain | - | - |
|  | 66.7% | 28.9% | 2.2% | 0% | 0% | 2.2% | - | - |
| If your recommendation as first-line is monotherapy with a non-platinum regimen, what agent would you recommend in an area with limited resources? | Paclitaxel | Topotecan | Gemcitabine | Vinorelbine | 5-FU | Ifosfamide | Best supportive care | Abstain |
|  | 71.6% | 7.5% | 9.0% | 1.5% | 7.5% | 0% | 0% | 3.0% |
| What is your first-line treatment for patients with metastatic or recurrent cervical cancer not amenable to salvage loco-regional treatment in areas without access to taxanes or where taxane-related costs are prohibitive? | Cisplatin and 5-fluorouracil | Cisplatin | Fluorouracil | None | Abstain | - | - | - |
|  | 61.8% | 27.3% | 5.5% | 0% | 5.5% | - | - | - |
| What is the recommended treatment option for patients with potentially resectable local recurrence without suspicion of lymph node involvement and no comorbidities who were submitted to prior surgery without adjuvant treatment in an area with limited resources? | Weekly cisplatin, 40 mg/m² IV with radiation therapy | Radiation therapy alone | Salvage surgery alone | Salvage surgery followed by radiation therapy | Salvage surgery followed by weekly cisplatin, 40 mg/m² IV with radiation therapy | Cisplatin-based therapy | Best supportive care | Abstain |
|  | 42.5% | 0% | 13.7% | 8.2% | 31.5% | 1.4% | 1.4% | 1.4% |
| What is the recommended treatment option for patients with potentially resectable local recurrence without suspicion of lymph node involvement who were submitted to prior surgery without adjuvant treatment, with comorbidities and/or not eligible to receive cisplatin and where radiotherapy is available? | Carboplatin with radiation therapy | Non-platinum agent with radiation therapy | Radiation therapy alone | Salvage surgery alone | Salvage surgery followed by radiation therapy and or chemoradiation | Chemotherapy alone | Best supportive care | Abstain |
|  | 30.0% | 4.3% | 30.0% | 8.6% | 25.7% | 0% | 1.4% | 0% |
| What is the recommended treatment option for patients with potentially resectable locally recurrent disease without suspicion of lymph node involvement who were submitted previously to surgery without adjuvant treatment and with comorbidities and/or not eligible to cisplatin in an area with limited resources where radiotherapy is not available? | Salvage surgery alone | Salvage surgery followed by carboplatin-based chemotherapy | Salvage surgery followed by non-platinum agent chemotherapy | Carboplatin-based chemotherapy followed by surgery | Carboplatin-based chemotherapy | Best supportive care | Abstain | - |
|  | 38.2% | 18.4% | 6.6% | 4.0% | 18.4% | 10.5% | 4.0% | - |
| What is the recommended treatment option for a resectable locoregional recurrence without suspicion of lymph node involvement in patients without comorbidities who were submitted previously to radiation therapy in an area with limited resources? | Cisplatin with re-radiation therapy | Re-radiation therapy | Salvage surgery alone | Salvage surgery followed by re-radiation therapy | Salvage surgery followed by chemotherapy | Platinum-based therapy | Best supportive care | Abstain |
|  | 0% | 0% | 48.7% | 5.3% | 32.9% | 7.9% | 1.3% | 4.0% |
| What is the recommended treatment option for a resectable locoregional recurrence without suspicion of lymph node involvement in patients with comorbidities and/or contra-indication to cisplatin who were submitted to radiation therapy in an area with limited resources? | Carboplatin with re-radiation therapy | Non-platinum agent with re-radiation therapy | Re-radiation therapy alone | Salvage surgery alone | Salvage surgery followed by re-radiation therapy +/- non-cisplatin chemotherapy | Systemic chemotherapy | Best supportive care | Abstain |
|  | 3.1% | 0% | 0% | 54.7% | 18.8% | 10.9% | 7.8% | 4.7% |
| If you do not recommend cisplatin as a radiosensitizing agent with radiation due to contra-indication, what is the recommended treatment option for a resectable locoregional lymph node recurrence what is your choice in an area with limited resources? | Carboplatin | Paclitaxel | Gemcitabine | 5-FU | I do not recommend any agent | Abstain | - | - |
|  | 70.7% | 6.9% | 3.5% | 5.2% | 6.9% | 6.9% | - | - |
| What is the recommended treatment option for a locoregional resectable lymph node recurrence in a patient without comorbidities treated initially only with surgery in an area with limited resources? | Chemoradiation with cisplatin | Radiation therapy | Salvage surgery alone | Salvage surgery followed by radiation therapy | Salvage surgery followed by weekly cisplatin, 40 mg/m² IV with radiation therapy | Cisplatin-based therapy | Best supportive care | Abstain |
|  | 59.1% | 3.0% | 1.5% | 15.2% | 19.7% | 1.5% | 0% | 0% |
| What is the recommended treatment option for a resectable locoregional lymph node recurrence in a patient without comorbidities treated initially with surgery and adjuvant radiation or chemoradiation (nodes not previoulsy irradiated) in an area with limited resources? | Cisplatin with re-radiation therapy | Re-radiation therapy | Salvage surgery alone | Salvage surgery followed by re-radiation therapy | Salvage surgery followed by weekly cisplatin or carboplatin and paclitaxel with re-radiation therapy | Systemic chemotherapy | Best supportive care | Abstain |
|  | 31.8% | 4.6% | 29.6% | 0% | 13.6% | 13.6% | 2.3% | 4.6% |
| What is the recommended treatment option for a resectable locoregional lymph node recurrence in a patient without comorbidities treated initially with surgery and adjuvant radiation or chemoradiation (nodes previoulsy irradiated) in an area with limited resources? | Cisplatin with re-radiation therapy | Re-radiation therapy | Salvage surgery alone | Salvage surgery followed by re-radiation therapy | Salvage surgery followed by weekly cisplatin or carboplatin and paclitaxel with re-radiation therapy | Systemic chemotherapy | Best supportive care | Abstain |
|  | 6.3% | 0% | 46.9% | 3.1% | 9.4% | 31.3% | 1.6% | 1.6% |
| What is the recommended second-line treatment for patients who failed platinum-based therapy in an area with limited resources? | Paclitaxel | Gemcitabine | 5-FU | Methotrexate | Best supportive care | Abstain | - | - |
|  | 49.2% | 35.6% | 3.4% | 0% | 6.8% | 5.1% | - | - |
| For women with metastatic cervical cancer previously treated and with no clinical trial available, when do you recommend best supportive care in an area with limited resources? | After first-line treatment | After second-line treatment | After third-line treatment or more | P*erformance status* > *2*, unrelated to line of treatment | Abstain | - | - | - |
|  | 11.1% | 24.1% | 11.1% | 51.9% | 1.9% | - | - | - |
| Would you consider testing PD-L1 status and/or microsatellite instability in metastatic cervical cancer? | Yes, in the majority of patients | Yes, in a minority of selected patients | No | Abstain | - | - | - | - |
|  | 33.9% | 35.5% | 27.4% | 3.2% | - | - | - | - |
| Would you consider metastasectomy, radiation therapy, or either for oligometastatic (< 4 lesions and restricted to one organ) cervical cancer (excluding bone metastasis) in an area with limited resources? | In the majority of patients, and I prefer surgery | In the majority of patients, and I prefer radiation | In a minority of patients, and I prefer surgery | In a minority of patients, and I prefer radiation | I consider both as equivalent | I do not recommend either | Abstain | - |
|  | 19.1% | 19.1% | 14.7% | 25.0% | 10.3% | 11.8% | 0% | - |
